# Solubility Limit and Optoelectronic Evolution of Heterovalent Bi^3+^ Doping in MAPbBr_3_ Perovskite Powders: A Study from Electrochemical Impedance Spectroscopy (EIS)

**DOI:** 10.3390/nano16201319

**Published:** 2026-10-19

**Authors:** Péyèna Yacouba Dao, Andreu Andrio, Júlia Marí-Guaita, Giulia Longo, Shafi Ullah, Donafologo Soro, Álvaro Montero Reguera, Bernabé Mari Soucase, Vicente Compañ

**Affiliations:** 1 Institut de Fabricació i Producció Automatitzada, Universitat Politècnica de València, Camí de Vera s/n, 46022 València, Spain; 2 Departament de Física, Universitat Jaume I, Avinguda Sos Baynat s/n, 12071 Castellón, Spain; 3 Département des Sciences et Technologie, École Normale Supérieure (ENS) d’Abidjan, Abidjan 08 BP 10, Côte d’Ivoire; 4 Departamento de Termodinámica Aplicada (ETSII), Universitat Politècnica de València, Camí de Vera s/n, 46022 València, Spainvicommo@ter.upv.es (V.C.)

**Keywords:** heterovalent doping, bismuth, solubility limit, electrochemical impedance spectroscopy, charge transport, dielectric properties

## Abstract

This paper reports a comprehensive physicochemical and electrical investigation of MAPb_1−x_Bi_x_Br_3_ perovskite powders (nominal x = 0 to 0.16) synthesized by antisolvent (acetone) precipitation. Structural (XRD/Rietveld) and chemical (EDX/EDS) analyses reveal a dual-regime doping mechanism governed by a thermodynamic solubility limit, with the true bismuth content saturating at approximately 1.3–1.4 at.% (nominal x ≈ 0.04). Below this limit, bismuth insertion causes anomalous lattice expansion through the creation of lead vacancies (V″_Pb_) and leads to a marked increase in the absolute intensity of the main diffraction peak, despite a slight broadening of the FWHM driven by local lattice microstrains. Above the limit, excess bismuth is expelled toward the grain boundaries as a highly disordered, amorphous-like, or short-range segregated secondary phase, degrading crystallinity and narrowing the effective optical band gap by up to 35 meV (from 2.278 eV to 2.243 eV) following an initial stabilization at low introduction levels (1–2% Bi), driven by the tentative formation of localized sub-gap defect states (Urbach-type tails) rather than intrinsic band-structure reorganization. Electrochemical impedance spectroscopy (EIS) confirms this dual regime: the absence of an electrode-polarization peak in ε″ indicates a Maxwell–Wagner–Sillars-type response, allowing the bulk conductivity to be extracted directly from the dielectric loss. At low temperatures, two distinct conduction regions coexist—a high-frequency electronic (hole-like) process and a low-frequency ionic process linked to bismuth-related defects, with ionic transport becoming dominant at high temperature; correspondingly, the dc-conductivity activation energy decreases from 66.3 kJ/mol (x = 0) to 30.7 kJ/mol (x = 0.16). Finally, the high-frequency limiting permittivity and geometric capacitance increase significantly with bismuth content (from ~15 pF to ~30 pF), reflecting enhanced dielectric performance driven by defect engineering.

## 1. Introduction

ABX_3_ hybrid organic–inorganic lead-halide perovskites have revolutionized the field of optoelectronics over the past decade, powered by outstanding performance in photovoltaic cells, light-emitting diodes (LEDs), and photodetectors [1]. Among this family of materials, methylammonium lead bromide MAPbBr_3_ is of major interest because of its direct optical gap in the visible range (~2.3 eV), its high absorption coefficients, its long charge-carrier diffusion lengths and its remarkable tolerance to structural defects [2]. Nevertheless, the large-scale deployment of these devices remains hampered by two major obstacles: the intrinsic instability of the organic phase under environmental stress and the environmental toxicity linked to the presence of divalent lead Pb^2+^ [3,4,5].

To overcome these limitations while modulating the electronic properties of the matrix, crystal engineering through heterovalent doping has emerged as a cutting-edge strategy [6]. The introduction of isomorphic trivalent cations, particularly bismuth Bi^3+^, proves to be particularly attractive. The Bi^3+^ cation has an ionic radius (1.03 Å) close to that of Pb^2+^ (1.19 Å), allowing for its substitutional integration at the B site of the perovskite lattice [7]. Electronically, this heterovalent doping is expected to induce n-type behavior through the addition of excess electrons, thus modifying the density of free carriers and the material’s conductivity [8,9,10,11].

However, the exact impact of incorporating bismuth into the 3D structure of MAPbBr_3_ still raises significant controversy in the scientific literature. While early studies reported an effective narrowing of the bandgap attributed to a modification of the valence and conduction bands [8,10,11], recent work rather suggests that this optical shift originates from the creation of a high density of deep defect states localized below the gap, commonly called Urbach tails [9]. Moreover, the effective solubility limit at which the perovskite lattice saturates and begins to expel the excess dopant, possibly in the form of disordered or amorphous-like secondary phases, remains poorly defined for macroscopic powder samples. Finally, the direct correlation between these local structural transitions and the dynamics of electrical charge transfer, classically probed by electrochemical impedance spectroscopy (EIS), has only been the subject of rare systematic investigations [12].

In this work, we present a comprehensive study of the physicochemical properties of a series of MAPb_1−x_Bi_x_Br_3_ powders synthesized with nominal bismuth levels ranging from 0% to 16%. By combining high-resolution X-ray diffraction (XRD) assisted by Rietveld refinement, electron microscopy coupled with quantitative EDX mapping, diffuse reflectance spectroscopy converted via the Kubelka–Munk function, and impedance spectroscopy (EIS), we highlight a dual-regime doping mechanism dictated by an effective solubility limit. Our results illuminate the fundamental boundary between the proper incorporation of the dopant into the lattice and surface microstructural segregation phenomena, paving the way for precise control of the optoelectronic properties of these next-generation materials. It should be noted that this work is a fundamental physicochemical investigation of macroscopic powders and compressed powder compacts; no thin films or optoelectronic devices were fabricated or characterized.

## 2. Experimental Section

### 2.1. Reagents

All chemicals were used as received, without further purification. Methylammonium bromide (MABr, ≥99%), lead (II) bromide (PbBr_2_, ≥98%), bismuth (III) bromide (BiBr_3_, ≥98%), N, N-dimethylformamide (DMF, ≥99.8%) and dimethyl sulfoxide (DMSO, ≥99.9%) were purchased from Sigma-Aldrich (Darmstadt, Germany).

### 2.2. Synthesis of MAPb_1−x_Bi_x_Br_3_ Perovskite Powders

A series of MAPb_1−x_Bi_x_Br_3_ powders with nominal bismuth concentrations of x = 0, 0.01; 0.02; 0.04; 0.08 and 0.16 were synthesized by an anti-solvent induced precipitation method at room temperature under ambient air conditions.

For the pure MAPbBr_3_ reference sample (x = 0), stoichiometric amounts of MABr (2 mmol) and PbBr_2_ (2 mmol) were dissolved in 2 mL of a DMF: DMSO (7:3 *v*/*v*) solvent mixture to obtain a clear precursor solution with a concentration of 1 M. For the Bi^3+^-doped samples, a portion of the PbBr_2_ was systematically replaced by BiBr_3_ to maintain a total metal cation concentration of 1 M, according to the following stoichiometric relationships:

(1) $$n\left(Bi{Br}_{3}\right)=x\cdot n_{total}=x\cdot 2\;mmol$$

(2) $$n\left(Pb{Br}_{2}\right)=\left(1-x\right)\cdot n_{total}=\left(1-x\right)\cdot 2\;mmol$$

where *x* represents the nominal mole fraction of bismuth (*x* = 0; 0.01; 0.02; 0.04; 0.08; 0.16) and *n*_total_ = 2 mmol is the total amount of substance of metallic cations in the 2 mL of solution.

The precursor solutions were kept under stirring for 12 h at 60 °C at a speed of 1200 rpm to ensure complete dissolution. Then, each solution was added dropwise into 20 mL of acetone under vigorous stirring, triggering the immediate precipitation of powders, changing from a bright orange to a dark brown hue. The obtained powders were dried in an oven for 24 h at 90 °C to prevent hydration and eliminate residual solvents.

### 2.3. Characterization Techniques

- Structural characterization: X-ray diffraction (XRD) patterns were recorded on a RIGAKU Ultima IV diffractometer (Tokyo, Japan) equipped with a Cu Kα radiation source (λ = 1.54 Å), operating at 40 kV and 40 mA. The data were collected over a 2θ range from 5° to 80° with a sampling step of 0.02° and a scanning speed of 2°/min. The quantitative lattice parameters and phase purity were determined by Rietveld refinement using the GSAS-II (General Structure Analysis System II) software (v5.6.0).
- Quantitative energy-dispersive X-ray spectroscopic (EDX) analyses as well as spatial elemental mappings (EDS mapping) were acquired using a ZEISS ULTRA 55 field emission scanning electron microscope (FESEM, Gemini technology; ZEISS, Oberkochen, Germany) equipped with an Ultim Max EDS detector (Oxford Instruments, Abingdon, UK), under an acceleration voltage of 20 kV. Spectra acquisition, quantification and mapping were carried out with AZtec 3.3 software (Oxford Instruments, Abingdon, UK). For the quantitative analyses, the spectra were acquired for 5 min per area and six different areas were analysed per sample. The quantification was carried out with standards, with a relative error of 5%, which corresponds to an uncertainty of approximately ±0.1 at.% for the measured Bi content. Since the analyses were performed on powder samples (rough, non-flat surfaces), the EDS atomic concentrations should nevertheless be regarded as semi-quantitative.
- Optical characterization: Diffuse reflectance (R) spectra were collected in the wavelength range of 300 to 800 nm using a high-resolution HR4000 spectrometer coupled with an LS-1-LL long-life tungsten halogen light source and a reflection probe (all from Ocean Optics, Orlando, FL, USA). A white PTFE diffuse reflectance standard (WS-1, Ocean Optics) was used as the reference during measurement. Spectra were acquired and processed with OceanView version 2.0 (Ocean Optics, Orlando, FL, USA).
- Electrochemical Impedance Spectroscopy: The powders of MAPb_1−x_Bi_x_Br_3_, synthesized to measure their electrical conductivity, were previously dried in a vacuum cell. Subsequently, the powders were pelletized using a 10 mm die mold set in a press under a pressure of 15 t/cm^2^ forming the measured disks, homogenized by following the same protocol for the fabrication of all of them. Then, the thickness of the pellets was determined using a micrometer, taking the average of five measurements in different surface planes. Next, the pellets were inserted between two gold disc electrodes of 10 mm diameters (S = 0.785 cm^2^) attached to the impedance spectrometer acting as blocking electrodes in a Novocontrol broadband dielectric Spectrometer (BDS, Concept 80, Hundsangen, Germany) combined with an SR 830 lock-in amplifier with an Alpha dielectric interface in the range of 10^−1^ to 10^7^ Hz, utilizing a 0.1 V signal amplitude to avoid any non-linear effects, in a range of temperature between 20 and 200 °C in steps of 20 °C, following the same procedure previously used by our group for undoped MAPbX_3_ perovskite powders [13]. The measured samples are therefore continuous, self-supporting compressed powder compacts (not thin films) with a well-defined geometry; their thicknesses were L = 512, 446, 669, 471, 459 and 575 μm for x = 0, 0.01, 0.02, 0.04, 0.08 and 0.16, respectively (Table S2 of the Supplementary Materials).

## 3. Results and Discussion

### 3.1. Structural Properties and Rietveld Refinement (XRD)

The phase purity, crystal symmetry, and structural evolution of the MAPb_1−x_Bi_x_Br_3_ powder series (*x* = 0 to 0.16) were studied by X-ray diffraction (XRD) on a Rigaku Ultima IV diffractometer, as illustrated in Figure 1. Analysis of the powder X-ray diffraction patterns demonstrates that the three-dimensional cubic framework of the material is preserved upon doping, although continuous bismuth incorporation introduces structural distortion and small changes in the lattice parameter [7]. While such modifications are often reported in related systems, our MAPb_1−x_Bi_x_Br_3_ powders maintain a strictly cubic *Pm*3^−^*m* space group over the entire doping range studied.

Furthermore, no spurious crystalline diffraction peaks associated with unreacted precursors, such as PbBr_2_ or BiBr_3_, are detected for *x* ≥ 0.02. This demonstrates the high phase purity of our samples, echoing the high-efficiency synthesis standards established by Saidaminov et al. [14] for pristine crystals where unreacted PbX_2_ traces are completely eliminated. The only exception is the *x* = 0.01 sample, whose diffractogram exhibits additional weak reflections (e.g., at 2 *θ* ≈ 18–19°) that cannot be indexed to the cubic perovskite phase. Their very low intensity relative to the (100) reflection indicates a trace amount of a minor secondary phase, most plausibly residual unreacted precursor or a solvate intermediate retained during the rapid antisolvent precipitation; its sporadic presence does not affect the structural analysis of the perovskite matrix. At high nominal rates (*x* = 0.08 and 0.16), the absence of sharp peaks attributable to the two-dimensional derived phase MA_3_ Bi_2_ Br_9_ suggests that the excess bismuth meaning that the excess bismuth may reside either as an amorphous-like network or within disordered clusters below the XRD coherent detection limit detectable by standard X-ray diffraction [15].

To probe the local geometric variations in the lattice, the lattice parameters were refined from the powder XRD patterns using the Rietveld refinement procedure implemented in GSAS-II. The refinements were intended to determine the evolution of the lattice parameter as a function of Bi content, while the crystal structure (*Pm*3^−^*m*) was kept fixed. The refined parameters included the scale factor, background, zero shift, lattice parameter, and selected profile parameters. To ensure the high fidelity of the structural parameters, a representative refinement profile for the pristine MAPbBr_3_ framework is displayed in Figure 2. The structural refinement converged successfully within the cubic Pm3^−^m space group, yielding a profile residual (R_p_) of 35.59%, a weighted profile residual (R_wp_) of 49.07% (precisely 49.066%) on 3751 observations, a phase residual (R_F_) of 65.06%, and an unweighted phase residual (R_F_^2^) of 73.96% on 24 reflections. The complete structural alignment is quantified by a total χ^2^ of 1.68741 × 10^7^, a reduced χ^2^ (Goodness of Fit) of 67.13 over 7 refined parameters, and a Durbin–Watson statistic of 0.036. Crucially, the statistical standard deviation (esd) computed from the metric tensor for the baseline cell parameter a is as low as ±0.00001 Å, giving an absolute value of 5.92173 ± 0.00001 Å. Although the profile residuals reflect typical localized microstructural strains and diffuse scattering contributions characteristic of rough, non-flat macroscopic powder compacts synthesized via rapid anti-solvent precipitation, the relatively high residual values indicate limitations of the present refinement and structural model; nevertheless, the refinement provides a consistent estimate of the lattice parameter for comparative analysis across the Bi-doped series.

The evolution of the lattice parameter *a* as a function of the nominal dopant concentration exhibits a highly nonlinear behavior, deviating from the classical Vegard law [16]. For the pure reference sample (*x* = 0), the lattice parameter is 5.92173 Å, a value in perfect agreement with fundamental data from the literature for MAPbBr_3_ [17]. Upon the introduction of 1% nominal bismuth (x = 0.01), a clear expansion of the bromide cage is observed, with parameter *a* increasing to 5.92664 Å. From a purely steric point of view, replacing lead with bismuth should induce a contraction of the lattice, the ionic radius of Bi^3+^ (≈1.03 Å) being smaller than that of Pb^2+^ (≈1.19 Å) [18]. The observed increase in the lattice parameter suggests that the incorporation of Bi involves a more complex mechanism than a simple substitution based solely on ionic radii. A highly plausible proposed mechanism is the formation of charge-compensating defects, such as lead vacancies (V″_Pb_) or organic cation modifications, as proposed in previous reports [8]. These lattice vacancies modify local Coulombic interactions and cause a relaxation of the [PbBr_6_]^4−^ octahedral cage, pushing bromide anions outwards [8]. From *x* = 0.02 to *x* = 0.16, the lattice parameter reaches an effective solubility plateau oscillating weakly between 5.92459 Å and 5.92633 Å. This saturation profile could indicate that the host crystal lattice reaches an apparent saturation limit for hosting the dopant cation within its coherent domains.

This dual-regime incorporation dynamic is quantitatively supported by the joint evolution of the full width at half maximum (FWHM) and the absolute maximum intensity of the main diffraction peak located at 2 *θ* ≈ 14.9° (crystal plane (100)), as detailed in Table 1. In the first regime, from *x* = 0 to *x* = 0.04, the absolute intensity continuously escalates from 559,117 a.u. to a maximum threshold of 1,218,042 a.u., confirming an enhancement in long-range structural order. Concurrently, the FWHM slightly broadens from 0.0934° to 0.1071°, reflecting local lattice microstrains induced by the heterovalent substitution. Beyond the critical 4% threshold, a complete reversal occurs: the peak intensity drops sharply to 928,579 a.u. (*x* = 0.08) and further minimizes to 884,521 a.u. (*x* = 0.16). This structural degradation is accompanied by a non-monotonic fluctuation of the FWHM, which decreases to 0.0888° (*x* = 0.08) before rising to 0.1001° (*x* = 0.16). This non-monotonic behavior indicates a complex convolution of instrument broadening, microstrains, and crystallite size fluctuations. It highlights that above the effective solubility limit, the expulsion of excess bismuth toward the grain boundaries generates microstructural disorder and reduces the overall crystalline volume fraction of pure perovskite.

### 3.2. Quantitative Chemical Analysis and Spatial Distribution (EDX and EDS Mapping)

To physically validate the structural evolution model derived from the XRD analysis, energy-dispersive X-ray (EDX) spectroscopic analyses were performed at a target accelerating voltage of 20 kV, as illustrated in Figure 3. This specific operating voltage ensures optimal excitation of the high-energy lines of heavy cations, namely the lead L-line and the bismuth L-line, guaranteeing highly reliable chemical quantification. Macroscopic quantitative measurements confirmed the existence of a strict effective solid solubility limit within the perovskite matrix.

In the first regime, corresponding to low introduction rates (*x* ≤ 0.02), the actual atomic concentration of bismuth increases linearly and stoichiometrically with the nominal concentrations introduced in the precursor solution, rising from ≈0.5 at. % for the 1% Bi sample to ≈1.0 at. % for the 2% Bi sample. Given the typical relative uncertainty of EDS quantification on polycrystalline powder samples (around 5%, corresponding to roughly ±0.1 at.% in this concentration range), the measured values are reported to one decimal place. A clear break in this linearity occurs at *x* = 0.04, where the incorporated bismuth content levels off at ≈1.2 at. %. For the high nominal concentrations in the series (*x* = 0.08 and), the amount of bismuth plateaus at ≈1.3 at. % and ≈1.4 at. %, respectively. The measured Bi contents for the *x* = 0.08 and *x* = 0.16 samples are very similar and, within the experimental uncertainty of the EDS measurements, do not show a significant statistical difference. These results demonstrate that the Bi incorporation tends to saturate at high nominal concentrations. Taken together with the XRD lattice parameter analysis, these results are consistent with an effective solid solubility limit of bismuth in the three-dimensional crystal lattice of MAPbBr_3_ saturates at approximately 1.3–1.4 at.%, although semi-quantitative EDX profiling alone cannot definitively separate true substitution from grain boundary encapsulation.

The high-resolution elemental spatial maps (EDS mapping) acquired on a ZEISS Gemini microscope and presented in Figure 4 provide direct morphological and compositional evidence of this two-regime chemical transition. At low concentrations (*x* = 0.01 and 0.02), the maps reveal a uniform spatial distribution of bismuth, which co-localizes with the lead and bromine signals without any local agglomeration. This excellent homogeneity at the micrometer scale is compatible with a model of interstitial or substitutional insertion of Bi^3+^ within the host perovskite lattice, correlating with the initial lattice parameter dilatation observed by XRD.

A higher-magnification map acquired on the 4% Bi sample confirms the absence of localized clusters or isolated precursor islands, suggesting that the network uniformly accommodates the dopant up to its maximum steric hindrance capacity. To ensure structural validation, the native size scale bars (ranging from 10 µm to 50 µm) are embedded individually directly beneath each top-row SEM micrograph in Figure 4. The middle-row EDS maps correspond to the lead (Pb) distribution, while the bottom-row EDS maps display the bismuth (Bi) distribution. Both rows represent magnified chemical views corresponding strictly to the localized fields enclosed within the white bounding boxes on their respective SEM images, allowing for a proportional visual and quantitative assessment across all samples.

Conversely, a substantial microstructural change occurs for samples above the solubility threshold (*x* = 0.08 and 0.16). Elemental mapping reveals the emergence of a heavily granular and fragmented microstructure, characterized by a multitude of small granules and a loss of smooth single-crystal morphology. The excess precursor, no longer able to integrate into the saturated crystal lattice framework, acts as a powerful growth inhibitor during rapid nucleation. This bismuth surplus segregates from the coherent crystalline grains towards highly disordered or amorphous-like grain boundaries, forming a non-crystalline phase that can be phenomenologically described as highly disordered or amorphous-like. These EDS maps visually validate the overall intensity decrease and structural relaxation detected by XRD, bridging the physical boundary between bulk chemical engineering and surface microstructural segregation.

### 3.3. Optical Properties and Band Gap Engineering

The absorption properties and electronic structure of the MAPb_1−x_Bi_x_Br_3_ powder series were evaluated at room temperature by ultraviolet–visible (UV-Vis) diffuse reflectance spectroscopy. The raw diffuse reflectance spectra, recorded in the 300–800 nm range, are shown in Figure 5A. For the pristine reference sample (0% Bi), the reflectance profile is characterized by a flat diffuse scattering plateau at high wavelengths (>550 nm), followed by an extremely abrupt intensity drop localized around 542 nm. This sharp cutoff is the typical signature of a direct interband transition within the MAPbBr_3_ host matrix. The gradual introduction of bismuth induces a visible shift in this absorption front toward lower energies, extending to nearly 560 nm for the 16% Bi sample.

To overcome the pure scattering effects inherent to macroscopic powdered samples, the raw reflectance data were converted into equivalent absorbance using the Kubelka–Munk mathematical transformation, *F*(*R*) = (1 − *R*)^2^/2*R*. The resulting absorption spectra, illustrated in Figure 5B, confirm the progressive shift in the absorption threshold. Remarkably, a distinct broadening and smoothing of the absorption slope is observed for the heavily doped samples (8% and 16% Bi). Instead of dropping vertically toward the baseline, the foot of these curves forms a pronounced “heel” or a persistent low-energy absorption trail between 545 nm and 580 nm. This broadening is consistent with the emergence of Urbach-type tails [9]. These localized sub-gap states are most probably related to the structural disorder and granular morphology highlighted by EDS mapping at high concentrations. In disordered or defective semiconductors, localized states below the band edge give rise to an exponential absorption tail described by the Urbach rule, α(E) ∝ exp[(E − E_0_)/E_U_], where E_U_ is the Urbach energy, which increases with the degree of structural or compositional disorder; the optical transitions involving these states (band-to-tail and tail-to-tail) are the defect-related transitions responsible for the sub-gap absorption. In the present series, the tail extends over roughly 0.14 eV below the band edge (≈545–580 nm) for the 8% and 16% Bi samples, whereas it is much less pronounced for the 0–2% Bi samples, which points to a progressive increase in the density of defect-related sub-gap states with Bi content. A quantitative determination of E_U_ from the exponential region of F(R) is limited in powders by baseline and scattering contributions; therefore, the conclusion is based here on the comparison of the absorption-edge profiles (Figure 5B), and the attribution to Urbach tails is a tentative interpretation.

To precisely quantify the values of these energy transitions, the associated Tauc plots were constructed by plotting the quantity (*F*(*R*)*E*)^2^ as a function of the photon energy (*E*), as shown in Figure 6. This method assumes a parabolic band structure model with a square-root dependence on photon energy above the bandgap for direct interband transitions. Linear extrapolation of the steep region to the *x*-axis allows for the determination of the effective optical bandgap (*Eg*) for each sample in the series. The numerical values extracted are recorded in Table 2. Because the presence of exponential sub-gap defect states (Urbach tails) rounds the absorption edge and impacts the visual selection of the linear extrapolation region, an explicit experimental error bar of ± 0.005 eV has been assigned to all determined bandgap values to ensure scientific reliability. The small variation in E_g_ should therefore be understood as a convolution of the fundamental transition and the absorption-edge broadening caused by defect tails.

Analysis of these quantitative data reveals an overall reduction in the effective optical bandgap of approximately 35 meV over the entire series. A slight increase is initially observed at low introduction rates (1–2% Bi), before a progressive decrease becomes dominant from 4% Bi onwards, with the *Eg* value dropping from 2.278 ± 0.005 eV for the undoped reference sample to 2.243 ± 0.005 eV for the 16% Bi sample. At high nominal doses (8% and 16% Bi), the interception of the Tauc line with the energy axis continues to shift toward lower energies (2.253 ± 0.005 eV and 2.243 ± 0.005 eV, respectively), even though the actual bismuth incorporation into the bulk lattice has reached its effective solid solubility threshold. This continuous shift indicates that increasing the excess nominal precursor induces a higher density of electronic defect states localized at the grain boundaries. Consequently, the extension of the absorption profile toward the lower-energy region is governed by interfacial defect engineering rather than by a continuous modification of the core intrinsic electronic band structure of the host core crystal [9].

### 3.4. Electrochemical Impedance Spectroscopy (EIS)

Measurements of the complex permittivity by electrochemical impedance spectroscopy (EIS) were carried out in order to investigate charge transport in pressed pellets of MAPb_1−x_Bi_x_Br_3_ powder samples. The nominal Bi concentrations were x = 0, 0.01, 0.02, 0.04, 0.08, and 0.16, respectively.

The experimental impedance data were analyzed in terms of the complex relative permittivity function:

(3) $$\varepsilon^{*}\left(\omega ,T\right)=\varepsilon^{\prime }\left(\omega ,T\right)-j\varepsilon^{\prime \prime }\left(\omega ,T\right)$$

where ε′(ω,T) is the real part of the relative permittivity, commonly referred to as the dielectric constant, and ε″ (ω,T) is the imaginary part, or dielectric loss factor. Here, j = (−1)^1/2^, ω = 2πf is the angular frequency, (f) is the frequency of the applied electric field, and (T) is the measurement temperature. These two quantities, ε′ and ε″, describe the ability of the material to store and dissipate electrical energy, respectively.

In many impedance studies of perovskite powder samples, well-defined dielectric-loss peaks are not observed at low and intermediate frequencies [13]. A similar behaviour is found here for the MAPb_1−x_Bi_x_Br_3_ powders. Nevertheless, at low temperatures, a weak dielectric relaxation appears to be present at higher frequencies, both in the undoped MAPbBr_3_ sample and in the Bi-doped samples.

The measured dielectric response contains contributions from both polarization processes and charge transport. At low and intermediate frequencies, the response may be strongly affected by interfacial or space-charge polarization, whereas the dielectric loss can also contain a contribution from electrical conduction. In heterogeneous or porous materials, such contributions are often discussed in terms of Maxwell–Wagner–Sillars-type polarization effects [19,20]. Under these conditions, the effective complex conductivity can be related to the complex permittivity as:

(4) $$\sigma^{*}(\omega ,T)=j\omega \varepsilon (\omega ,T)$$

where ε_0_ is the vacuum permittivity [21]. Combining Equations (3) and (4) gives

(5) $$\sigma^{\prime }(\omega ,T)=\omega e_{0}\varepsilon^{\prime \prime }\left(\omega ,T\right)$$

and

(6) $$\sigma \prime \prime (\omega ,T)=\omega e_{0}\varepsilon^{\prime }\left(\omega ,T\right)$$

where σ′ and σ″ are the real and imaginary parts of the complex conductivity, respectively [22].

When electrode polarization produces a clear peak in ε″, separating the intrinsic conductivity from interfacial effects can be difficult. In the present MAPb_1−x_Bi_x_Br_3_ powders, however, no clear electrode-polarization peak is observed in ε″. This makes it more reasonable to estimate the conductivity from the dielectric-loss spectra, particularly in frequency ranges where the loss follows the behavior expected for ohmic conduction [23].

Figure 7 shows representative frequency dependences of ε′, ε″ and Tan δ for MAPb_1−x_Bi_x_Br_3_ with x = 0.04 at four selected temperatures (40, 80, 120, and 200 °C). The spectra show a relaxation-like decrease in ε′, a corresponding maximum in Tan δ, and a strong temperature-dependent dielectric loss over a broad frequency range. Similar behavior is observed for the other compositions.

In the frequency range where the dielectric loss is dominated by dc-conductivity, the following relation applies:

(7) $$\varepsilon \prime \prime =\frac{\sigma_{dc}}{2\pi \varepsilon_{0}f}$$

or, equivalently,

(8) $$log\varepsilon^{\prime \prime }=log\frac{\sigma_{dc}}{2\pi \varepsilon_{0}}-logf$$

Thus, a straight line with slope close to −1 in a log(ε″) versus log(f) plot indicates a conductivity-dominated response. The intercept of this line can then be used to estimate the dc conductivity, σ_dc_.

At low temperatures, the spectra suggest the presence of two frequency-dependent transport regions: one at higher frequencies and another at low-to-intermediate frequencies. These regions may reflect different charge-transport processes, possibly involving both electronic and ionic contributions. With increasing temperature, the low-frequency conductivity contribution becomes more pronounced and tends to dominate a wider frequency range.

The slope of the *log ε*″ versus *log f* curves also changes with frequency. In our Bi-doped MAPbBr_3_ perovskite powders, at frequencies below the maximum in Tan δ (*f* < *f_max_*_,*Tan*_*_δ_*) the slope is close to −1, which is consistent with a regime dominated by dc-like, long-range conduction [13]. At frequencies above this maximum (*f* > *f_max_*_,*Tan*_*_δ_*), the slope becomes less negative, with values close to −0.8 in some cases, indicating a more dispersive, hopping-type transport process in agreement with Jonscher’s universal power-law response [24]. It should be noted, however, that the exact slope values and the position of this crossover are sample-dependent and may be influenced by extrinsic factors such as impurity content, defect density, and electrode polarization. In particular, the accumulation of mobile ions near the electrodes can modify the low-frequency response and contribute to the observed conduction behaviour in these samples.

A closer inspection of Figure 7 shows that, for all four selected temperatures, namely 40, 80, 120, and 200 °C, the loss tangent Tan δ exhibits a well-defined maximum. This maximum shifts toward higher frequencies as the temperature increases. This behaviour indicates that the relaxation process becomes faster at higher temperature, as expected for thermally activated charge transport.

The position of the Tan δ maximum is also useful for analyzing the conductivity response. At higher temperatures, especially above 80 °C, the peak is better resolved within the experimental frequency window, allowing a more reliable interpretation of the conductivity data. At lower temperatures, the conductivity is weaker and the characteristic relaxation may be less clearly separated from the low-frequency response. Therefore, conductivity values estimated from Bode plots at low temperature should be treated with more caution.

Because the measurements were performed on pressed powder compacts placed between ion-blocking gold electrodes, the measured response is a convolution of several contributions that cannot be fully separated with the present data. (i) Intragrain (bulk) transport: both electronic carriers and mobile ionic species contribute to the conductivity; halide vacancies and, to a lesser extent, MA^+^ cations are the ionic species usually considered mobile in lead-halide perovskites [25]. (ii) Grain boundaries and particle–particle interfaces: in a compact, the charge carriers must cross numerous interfaces and pores, which gives rise to interfacial (Maxwell–Wagner–Sillars) polarization and can dominate the response at intermediate and low frequencies; the fragmented microstructure observed, and the Bi-rich disordered regions inferred, above the effective solubility limit may enhance this contribution. (iii) Electrode effects: since the gold electrodes block the ionic flux, mobile ions accumulate at the electrode/compact interfaces (electrode polarization), which affects the low-frequency permittivity and conductivity, particularly at high temperature. (iv) Contact and packing: the quality of the contact between the pellet and the electrodes, its porosity and its thickness influence the absolute values of the measured quantities. For these reasons, the conductivity σ_dc_ and the activation energies discussed below are effective (apparent) values of the whole compact and not intrinsic properties of the crystal lattice, and their dependence on the Bi content reflects the combined effect of changes in the intragrain defect chemistry and in the microstructure. Separating these contributions would require, for example, equivalent-circuit analysis of the impedance data, measurements on compacts of different thickness, electrodes with different ion-blocking character, or DC polarization experiments.

According to the electrode-polarization model described in Refs. [26,27], the frequency of the maximum in the loss tangent, *f_max_*_,*Tan*_*_δ_*= 1/2πτ_m_, is related to the relaxation time of the bulk material, τ, and to the electrode-polarization relaxation time, *τ_EP_*, by

(9) $$f_{max,Tan\;\delta }=\frac{1}{2\pi \sqrt{\tau \cdot \tau_{EP}}}$$

where τ and τ_EP_ are defined by

(10) $$\tau =\frac{\varepsilon_{0}\varepsilon_{\infty }}{\sigma_{dc}}$$

and

(11) $$\tau_{EP}=\frac{\varepsilon_{0}\varepsilon_{EP}}{\sigma_{dc}}$$

respectively, ε_∞_ being the permittivity determined at frequencies tending to infinity (f ⟶ ∞), ε_EP_ the permittivity at the electrode polarization, σ_dc_ the direct current conductivity, and ε_0_ the vacuum permittivity (i.e., 8.854 × 10^−12^ F/m). The value of ε_EP_ can be obtained when the low-frequency part of the ε′ spectrum approaches a plateau as f tends to zero. Alternatively, when electrode polarization produces a clear peak in ε″, the characteristic frequency of this peak can be related to the electrode-polarization relaxation time through f_max,ε″_ = 1/(2πτ_EP_) [28]. On the other hand, at high frequency (between 10^4^ Hz and 10^7^ Hz, depending on temperature), the real part of the permittivity is practically constant, independent of frequency. This plateau represents the high-frequency limiting permittivity, ε_∞_. This value represents the dielectric permittivity of our perovskites doped with Bi. In Figure 8 we can see the variation with temperature of permittivity, ε′(f ⟶ ∞), of the powder samples, collected in this study at f = 10^6^ Hz.

From Figure 8 we can see that the high-frequency limiting relative permittivity, ε_∞_, is practically constant between 20 °C and 80 °C, while it increases with temperatures above 80 °C, in all the samples. This temperature-induced increase is more pronounced in the Bi-doped samples, especially for the compositions with higher Bi content. For example, in the interval 20–100 °C the average relative dielectric constant ε′_∞_ takes the values 10.0 ± 0.1, 12.5 ± 0.2, 17.8 ± 0.5, 11.6 ± 0.1, 18.8 ± 0.2 and 21.8 ± 0.2 for the MAPb_1−x_Bi_x_Br_3_ (x = 0, 0.01, 0.02, 0.04, 0.08 and 0.16), respectively. At temperatures higher than 100 °C the permittivity continues to increase with the temperature, and this increase becomes stronger with higher Bi doping.

A close observation of Figure 8 shows that the values of the dielectric constant increase gradually with temperature up to 100 °C, above which the increase is much stronger and becomes more pronounced for higher Bi contents. On the other hand, in agreement with Figure 7, we observe that temperature variation in dielectric constant is more prominent at lower frequencies than at higher ones. At higher frequencies, the dipoles can no longer reorient themselves along the applied field. The dielectric relaxation behavior can then be due to a combination of different polarization mechanisms: interfacial polarization and space-charge polarization. Both depend on the charge diffusion responsible for the dipoles and on the relaxation time required for the displacement of the charges from the anode to the cathode.

Finally, as we mentioned above, the plots of loss permittivity ε″ vs. frequency shown in Figure 7 show a linear region in logarithmic scale. Such behaviour is observed not only in the 4% Bi sample; similar variations were observed for all the doping levels. Following the variation in loss permittivity ε″ vs. frequency and taking into account Equation (11), we could determine the dc-conductivity of the powders.

Figure 9 shows the variation in σ′ and Tan δ as a function of the frequency at different temperatures, for MAPb_1−x_Bi_x_Br_3_ with 0 ≤ x ≤ 0.16.

Figure 9 shows the Bode plots of the real part of the conductivity and the Tan δ of the samples MAPb_1−x_Bi_x_Br_3_ (x = 0, 0.01, 0.02, 0.04, 0.08 and 0.16) at five temperatures. A close inspection of this figure reveals that the real part of the conductivity increases with the frequency to then reach an almost constant value. This plateau corresponds to the dc conductivity, σ_dc_. The plateau becomes clearly identifiable when the phase angle approaches zero, indicating that the electrical response is mainly resistive. This condition occurs close to the frequency at which Tan δ reaches its maximum.

Therefore, the frequency region where σ′ becomes nearly independent of frequency, together with the position of the Tan δ maximum, was used as the criterion for estimating σ_dc_. The σ_dc_ values obtained for all powder samples are summarized in the Arrhenius plot shown in Figure 10.

Figure 10 depicts the dependence of the dc-conductivity of the powders at different concentrations of doping agent Bi with the temperature, where the conductivity decreases with decreasing temperature for all MAPb_1−x_Bi_x_Br_3_ powders, with apparent activation energies following the trend: E_act_ (x = 0) = (66.3 ± 1.8) kJ/mol > E_act_ (x = 0.01) = (61.2 ± 1.6) kJ/mol > E_act_ (x = 0.02) = (41.0 ± 1.5) kJ/mol > E_act_ (x = 0.04) = (40.2 ± 1.1) kJ/mol > E_act_ (x = 0.08) = (32.1 ± 0.6) kJ/mol > E_act_ (x = 0.16) = (30.7 ± 0.5) kJ/mol.

The activation energy obtained from the Arrhenius plot of the conductivity in Figure 10, σ_dc_ = σ_0_ exp(−E_a_/RT), is the energy barrier that the dominant mobile charge carriers must overcome, by thermally activated hopping between neighboring sites, to produce long-range transport across the compact. In lead-halide perovskites, the charge carriers involved are electronic carriers and mobile ions (mainly halide vacancies and, to a lesser extent, MA^+^ cations) [25]. Since the conductivity is determined for the whole compact, treating the entire system as a grain region, E_a_ is an apparent activation energy that includes intragrain, grain-boundary and interfacial contributions. The values found (0.69 eV for the undoped compact, decreasing to 0.32 eV for x = 0.16) are of the same order of magnitude as those usually reported for ion migration in lead-halide perovskites [25], which suggests that thermally activated hopping of charged species over potential barriers is the origin of the conduction mechanism in our powders; the decrease in E_a_ with Bi content means that the effective barrier for charge transport is lowered upon doping.

However, a detailed investigation of the conductivity values across the entire temperature range for MAPb_1−x_Bi_x_Br_3_ powders could be interpreted in terms of two different types of Arrhenius behavior: one at low temperatures (in a temperature range that depends on the Bi content), and another for high temperatures, such as is observed in Figure S1, where two activation energies are obtained. (See Supplementary Materials.)

Therefore, the above the activation energy will be correspond to the average value, where we can observe that ionic conductivity at low and moderate temperatures depends on the dopant agent content (% Bi). These perovskites doped with Bi are thermally activated and a change in relaxation frequency is observed by varying temperature. The average Arrhenius plot shows that dc-conductivity increases with the increasing temperature, which depicting a decrease in resistivity of the doped perovskite. Therefore, the apparent activation energy of perovskites without doping have higher activation energy that doped perovskites, these deceasing of E_act_ with the %Bi might be explained by two factors: first may be attributed to increasing the concentration of Bi increasing the diffusion of electrons increasing the amount of oxygen vacancies producing an increase in mobility and then an increasing of the conductivity. Secondly, might be ascribed to the lattice distortion or defects owing to the Bi substitution into Br.

The two Arrhenius regimes shown in Figure S1 (Table S1) indicate that more than one process contributes to the conductivity. Between 20 and 80 °C the apparent activation energies are low (12.5–43.2 kJ/mol, i.e., 0.13–0.45 eV), whereas between 100 and 200 °C they are higher (27.4–86.8 kJ/mol, i.e., 0.28–0.90 eV), and in both ranges they decrease with increasing Bi content. The lower barriers at low temperature suggest that less strongly activated processes (e.g., electronic hopping or transport along interfaces) are involved there, whereas the higher barriers at high temperature are more compatible with the migration of ionic defects, in agreement with the dominance of the low-frequency (ionic-like) contribution at high temperature mentioned above. Hall-effect measurements, which would give the carrier type, the carrier concentration and its temperature dependence, were not carried out in this work; therefore, the electronic/ionic assignment of the conduction processes and the n-type character expected for Bi^3+^ doping remain tentative.

Using the peak frequencies obtained from the Tan δ spectra, together with the dc conductivity values extracted from the Bode plots and the high-frequency limiting permittivity values, ε_∞_, the characteristic relaxation times using Equation (10) were calculated for the different MAPb_1−x_Bi_x_Br_3_ compositions. The values obtained can be observed in Figure 11.

As shown in Figure 11, the charge relaxation time during the diffusion process through the perovskite powders follows—as expected—the same Arrhenius behavior observed in the conductivity of the MAPb_1−x_Bi_x_Br_3_ powders, but with the sign reversed. When the system is considered as a grain region, the calculated apparent activation energy should be the same as that obtained for conductivity, but with the opposite sign, as can be seen by comparing Figure 10 and Figure 11; given that the relaxation time values plotted here for each temperature in the perovskites, represent the ratio of the matrix dielectric constant ε to the direct current conductivity σ_dc_. The variation in the apparent total relaxation time with 1000/T (K^−1^) follows a distribution of activation energies: *E*_act_ (x = 0 Bi) = 64.7 kJ mol^−1^ = 0.67 eV > *E*_act_ (x = 0.01 Bi) = 47.6 kJ mol^−1^ = 0.49 eV, *E*_act_ (x = 0.02 Bi) = 35.9 kJ mol^−1^ = 0.37 eV, *E*_act_ (x = 0.04 Bi) = 38.2 kJ mol^−1^ = 0.40 eV, *E*_act_ (x = 0.08 Bi) = 21.4 kJ mol^−1^ = 0.22 eV, *E*_act_ (x = 0.16 Bi) = 25.3 kJ mol^−1^ = 0.26 eV. From these results we observe that the activation energy associated with the total apparent relaxation time of the undoped perovskite is higher than that of the Bi-doped samples. The decrease in activation energy with bismuth content might be explained considering at least two factors. First, Bi doping may modify the concentration of vacancies or other point defects, thereby facilitating charge transport. Second, the incorporation of Bi into the perovskite lattice may produce local lattice distortion, which can alter the potential barriers for charge motion. The obtained activation energies therefore suggest that the charge diffusion process is thermally activated and may involve hopping of charged species over potential barriers. From this time, we can determine the apparent diffusion coefficient knowing the thickness of the sample sandwiched between the electrodes (L) by means of expression D = L^2^/τ. On the other hand, from the peak frequency value of tan δ, we can determine the electrode polarization relaxation time, τ_EP_, since

(12) $$\tau_{EP}=\frac{\tau_{m}^{2}}{\tau }$$

where τ_m_ = 1/2πf_m_, being f_m_ the frequency at which Tan δ reaches a maximum at each temperature. The values found for τ_EP_ and τ_m_ of powders MAPb_1−x_Bi_x_Br_3_ (x = 0, 0.01, 0.02, 0.04, 0.08, and 0.16 of Bi) are given in Table S2 of Supplementary Information.

Finally, the apparent capacitance of all the powders was calculated considering a parallel electrode geometry with d/A << 1 and negligible electrodes thickness, through the equation

(13) $$C(\omega ,T)=\frac{A}{L}\varepsilon_{0}\varepsilon_{r}(\omega ,T)$$

where *A* is the electrode area and *L* is the thickness of the powders, ε_0_ is the permittivity of free space or vacuum (8.854188 × 10^−12^ F/m) and ε_r_ the relative permittivity of the perovskite powders.

The values obtained for the apparent capacitance modulus (in pF) are plotted in Figure 12, in the frequency range where the phase angle approaches −90°, corresponding to a predominantly capacitive response, so that C ≈ |C|.

A close inspection of this plot shows two temperature regimes. Below 100 °C the capacitance has a constant value for each sample while above 100 °C the capacitance increases slightly with temperature. The apparent geometrical capacitance also changes with Bi concentration; however, as discussed below, this change cannot be attributed exclusively to Bi incorporation, because the apparent capacitance of a powder compact also depends on its geometry and packing.

As shown in Figure 12, the apparent geometrical capacitance increases with Bi incorporation. Even a small Bi content of x = 0.01 produces an increase of approximately 6 pF compared with the undoped sample. For higher Bi contents, the increase becomes more pronounced; for example, the sample with x = 0.08 shows an increase of about 15 pF.

Below approximately 160 °C, the capacitance follows the general trend:

(14) C(x=0)<C(x=0.01)≈C(x=0.02)≈C(x=0.04)<C(x=0.16)<C(x=0.08)

The average capacitance is approximately 14 pF below 100 °C for the undoped MAPbBr_3_ sample. For the lightly doped samples, namely x = 0.01, 0.02, and 0.04, the capacitance increases to around 19 pF; while for the samples with higher Bi content, x = 0.08 and x = 0.16, the capacitance reaches values of about 29 and 26 pF, respectively (Figure 12).

These results confirm that Bi incorporation enhances the capacitive response of the MAPb_1−x_Bi_x_Br_3_ powders. The apparent geometrical capacitance values obtained in this work for MAPb_1−x_Bi_x_Br_3_ (x = 0, 0.01, 0.02, 0.04, 0.08 and 0.16 of Bi) are higher than those reported for LaCrO_3_ and Ir-doped LaCrO_3_ systems [26,29].

Previous studies on Bi-doped MAPbBr_3_ have been carried out mainly on single crystals grown from solution [7,8,9,10,11,12] and have focused on band-gap and carrier-type engineering, photophysical properties, and transport or stability. Narrowing of the band gap was reported in early studies [8,10,11], whereas Nayak et al. [9] attributed the apparent reduction in the optical gap to sub-gap (Urbach-type) states rather than to a true band-structure modification, which is consistent with the tail-related gap narrowing (35 meV) observed here. Structural modifications induced by Bi doping in MAPbBr_3_ crystals were examined by Youn et al. [7], and the ionic conductivity and negative capacitance of Bi^3+^-doped perovskite single crystals were studied by Mansoorie et al. [12]. More recently, Wang et al. [30] investigated Bi^3+^ in a different perovskite-type lattice, the oxide double perovskites Ca_2_(La_1−x_Y_x_)NbO_6_, and showed by aberration-corrected TEM that Bi^3+^ occupies both the A and the B sites; the experimental optical band gap of these hosts decreases slightly upon Bi^3+^ incorporation (from 4.27 to 4.16 eV and from 4.26 to 4.22 eV), DFT calculations show Bi-derived states close to the valence-band maximum, and the degree of A-site order was found to control the structural rigidity and, thereby, the non-radiative recombination and the photoluminescence efficiency. Although these oxide phosphors differ from halide perovskites in composition, band-gap range and purpose (Bi^3+^ acts there as a luminescent activator), they illustrate qualitatively that, in perovskite-type lattices, the site occupancy and the degree of structural order around Bi^3+^ can strongly influence the electronic and optical response, a trend that is in line with the link proposed here between structural disorder (Bi-rich regions above the solubility limit) and sub-gap states. Compared with these works, the present study (i) deals with polycrystalline powders prepared by antisolvent precipitation, (ii) covers a wide range of nominal compositions (up to x = 0.16) and identifies, by combining XRD and EDX, an effective Bi solubility limit of about 1.3–1.4 at.%, above which the excess Bi probably segregates as a highly disordered or amorphous-like secondary phase, and (iii) correlates these structural regimes with the broadband dielectric/impedance response (σ_dc_, relaxation time and activation energies) between 20 and 200 °C. The comparison with single-crystal reports is qualitative, since the transport properties of compacted powders also include grain-boundary and interfacial contributions.

## 4. Conclusions

In conclusion, a direct correlation between local structural transitions, optical response, and charge transport dynamics was successfully established for the MAPb_1−x_Bi_x_Br_3_ system. Rietveld refinement of the XRD patterns revealed a markedly non-Vegard incorporation behavior: despite the smaller ionic radius of Bi^3+^ compared with Pb^2+^, the lattice parameter expands from 5.92173 Å for the undoped sample to 5.92664 Å at x = 0.01, pointing to the formation of charge-compensating defects such as lead vacancies, before stabilizing on a saturation plateau (≈5.925–5.926 Å) from x = 0.02 onwards. The joint evolution of the intensity and FWHM of the (100) diffraction peak identifies x ≈ 0.04 as the apparent framework saturation threshold, beyond which the excess bismuth is suggested to be expelled from the coherent diffraction domains and as a highly disordered secondary phase localized at the grain peripheries.

This structural picture is fully consistent with the optical response. Diffuse reflectance spectroscopy combined with the Kubelka–Munk analysis revealed a systematic red-shift in the absorption edge with increasing bismuth content, with the effective optical band gap extracted from the Tauc plots decreasing from 2.278 eV for the undoped reference to 2.243 eV for the nominally 16% doped sample, an overall reduction of 35 meV. After a slight widening of the gap at low doping levels (1–2% Bi), the narrowing becomes dominant from 4% Bi onwards and, beyond the structural saturation threshold, is accompanied by the emergence of Urbach tails. This tentatively indicates that the apparent gap reduction at high nominal concentrations originates from localized sub-gap defect states at the grain peripheries rather than from a continuous modification of the intrinsic band structure of the host crystal.

Heterovalent doping with Bi^3+^ also profoundly alters the perovskite’s electrical profile, lowering the dc conductivity (*σ_dc_*) activation energy from 66.3 kJ mol^−1^ (0.69 eV) at x = 0 to 30.7 kJ mol^−1^ (0.32 eV) at x = 0.16. This decrease is intrinsically linked to the reduction in the relaxation-time activation energies, attributed to the proliferation of crystalline vacancies and local lattice distortions. Furthermore, doping optimizes the dielectric properties by nearly doubling the material’s average geometric capacitance from 15 pF to 30 pF. These results validate a transport mechanism governed by charge carriers hopping over potential barriers. Together, the structural, optical, and electrical findings draw a clear line between volumetric electronic engineering and surface microstructural segregation phenomena, providing rigorous control keys for the design of high-performance and stable perovskite-based optoelectronic devices.

## Figures and Tables

**Figure 1:**
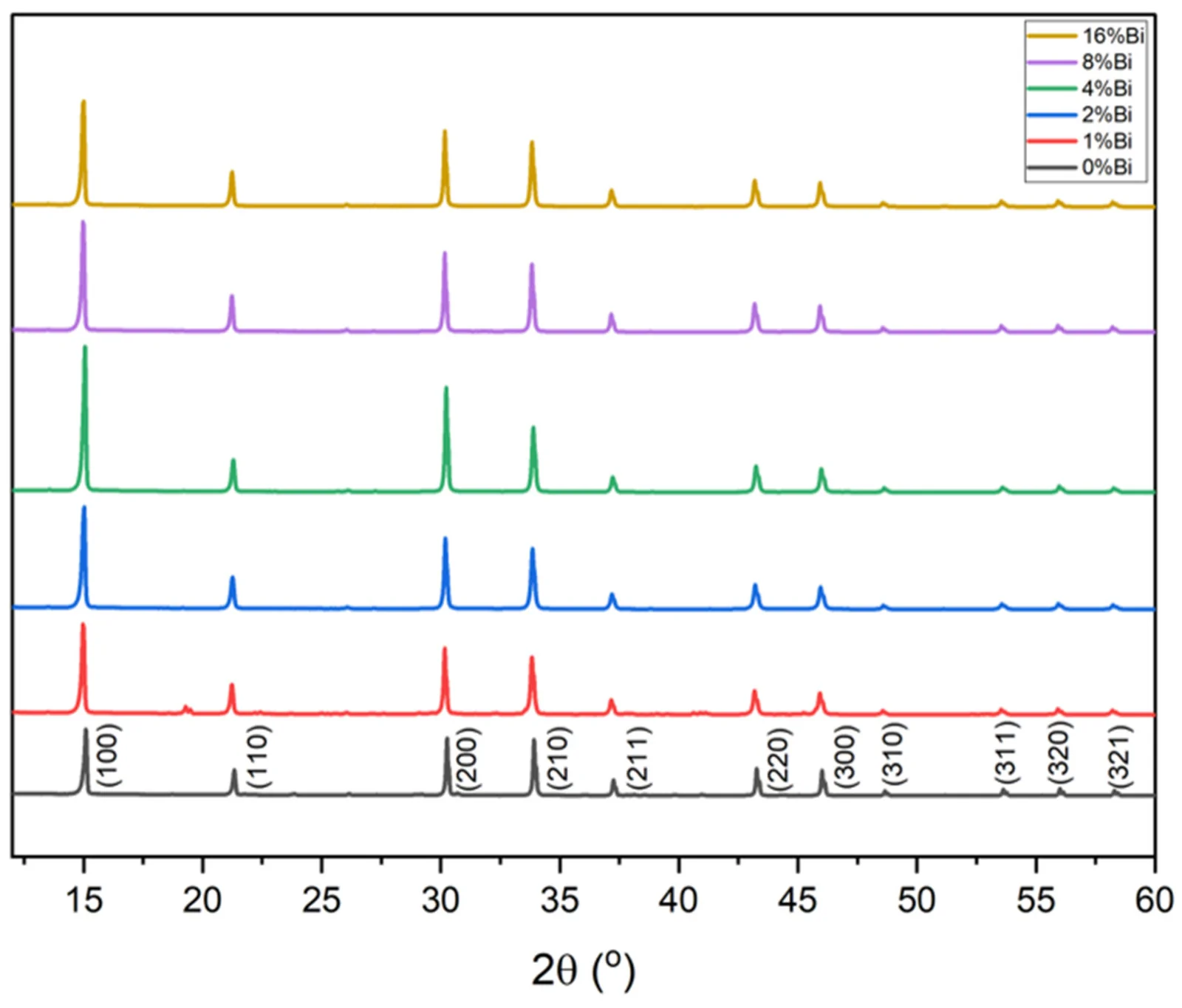
Experimental X-ray diffraction (XRD) patterns of MAPb_1−x_Bi_x_Br_3_ powders for nominal bismuth concentrations from 0% to 16%.

**Figure 2:**
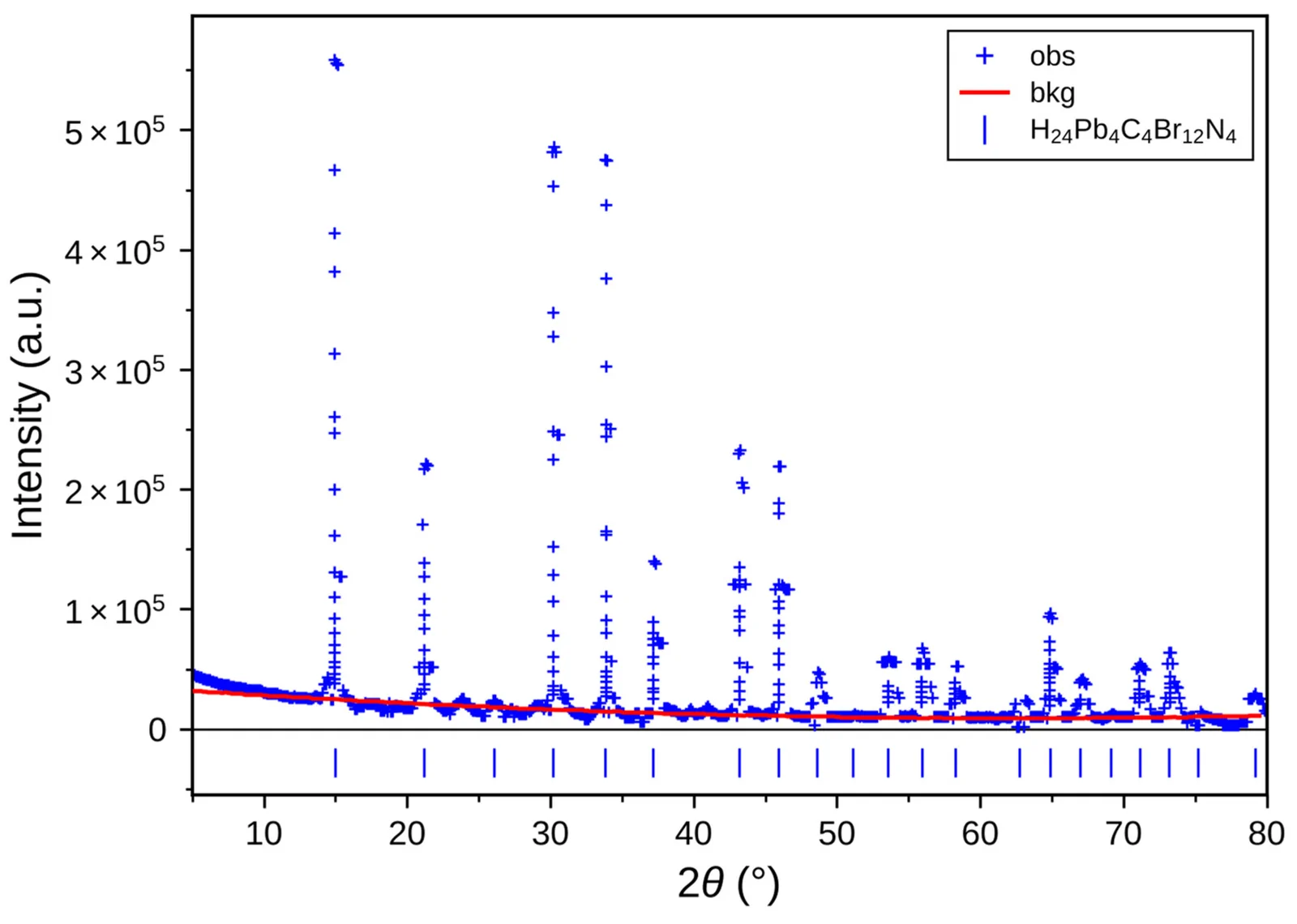
Rietveld refinement plot of the pristine MAPbBr_3_ powder XRD pattern performed in GSAS-II within the cubic *Pm*3^−^*m* space group. The blue crosses (obs) represent the experimental data points and the solid red line (bkg) represents the fitted background. The vertical blue ticks represent the allowed Bragg reflection positions for the H_24_Pb_4_C_4_Br_12_N_4_ phase. The structural fit converged with reliability residuals of R_p_ = 35.59%, R_wp_ = 49.07%, a reduced χ^2^ (Goodness of Fit) of 67.13, and a refined lattice parameter of a = 5.92173 ± 0.00001 Å.

**Figure 3:**
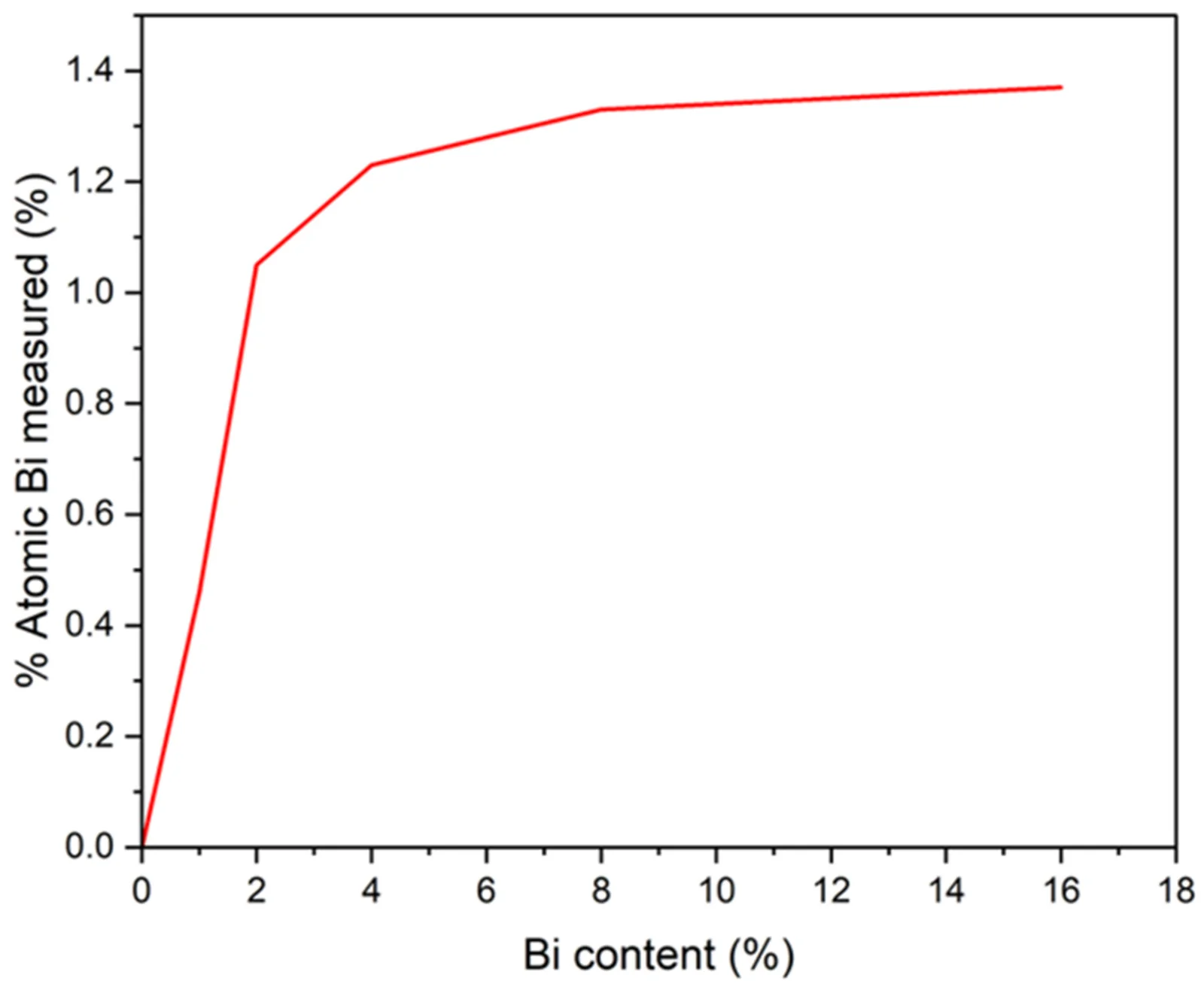
Evolution of the actual atomic concentration of bismuth measured by EDX spectroscopy (at 20 kV) as a function of the nominal doping rate (x) in the MAPb_1−x_Bi_x_Br_3_ series, highlighting the effective saturation plateau above 4%.

**Figure 4:**
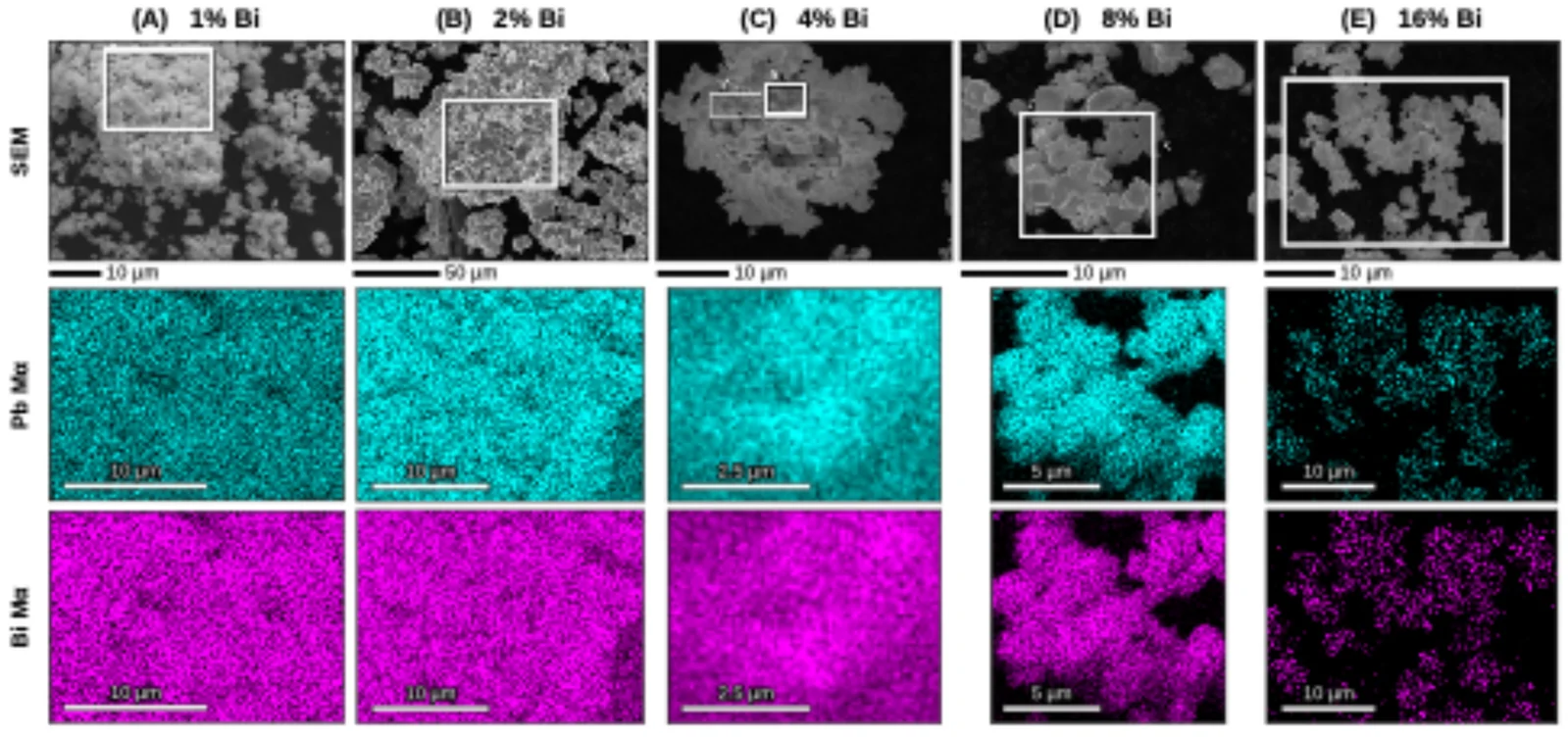
Scanning electron microscopy (SEM) micrographs (**top row**) and the corresponding energy-dispersive X-ray spectroscopy (EDS) elemental maps of Pb (Pb Mα, **middle row**) and Bi (Bi Mα, **bottom row**) of MAPb_1−x_Bi_x_Br_3_ perovskite powders with (**A**) 1% Bi, (**B**) 2% Bi, (**C**) 4% Bi, (**D**) 8% Bi and (**E**) 16% Bi. Each EDS map shows the area enclosed by the white box in the SEM micrograph above it. The native size scale bars are embedded individually directly beneath each image.

**Figure 5:**
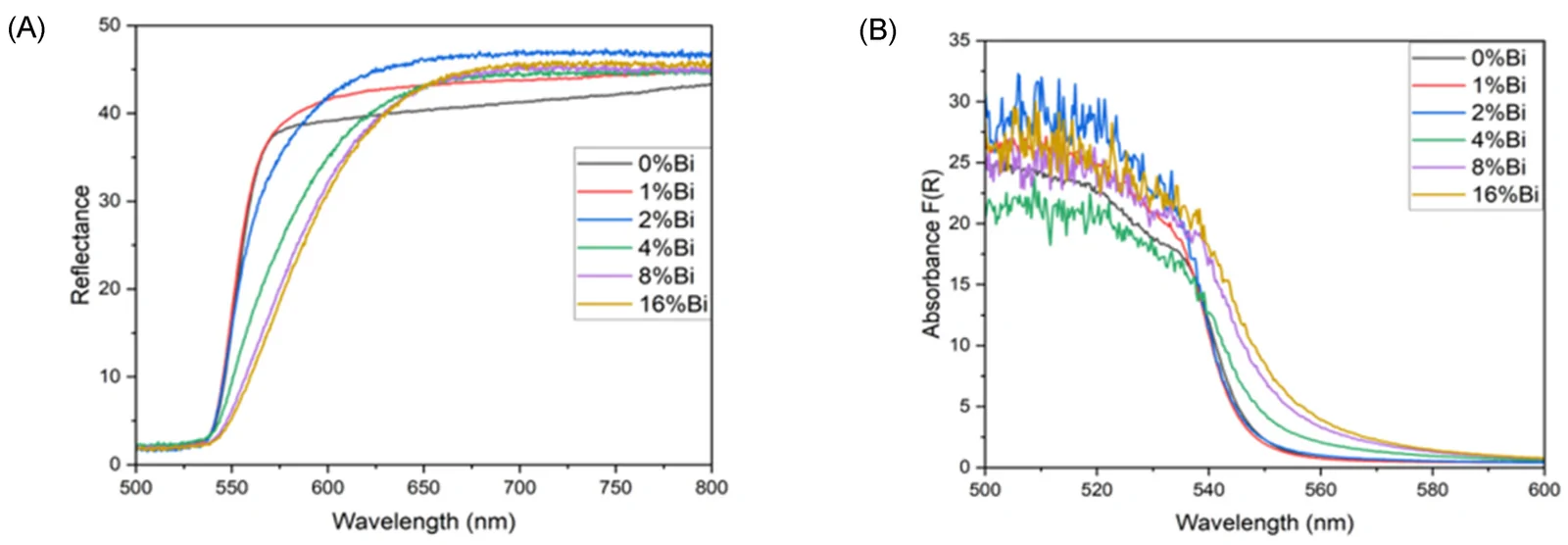
Optical characterizations of the MAPb_1−x_Bi_x_Br_3_ powder series as a function of wavelength: (**A**) Raw diffuse reflectance (*R*) spectra and (**B**) corresponding equivalent absorption spectra obtained by the Kubelka–Munk transformation *F* (*R*).

**Figure 6:**
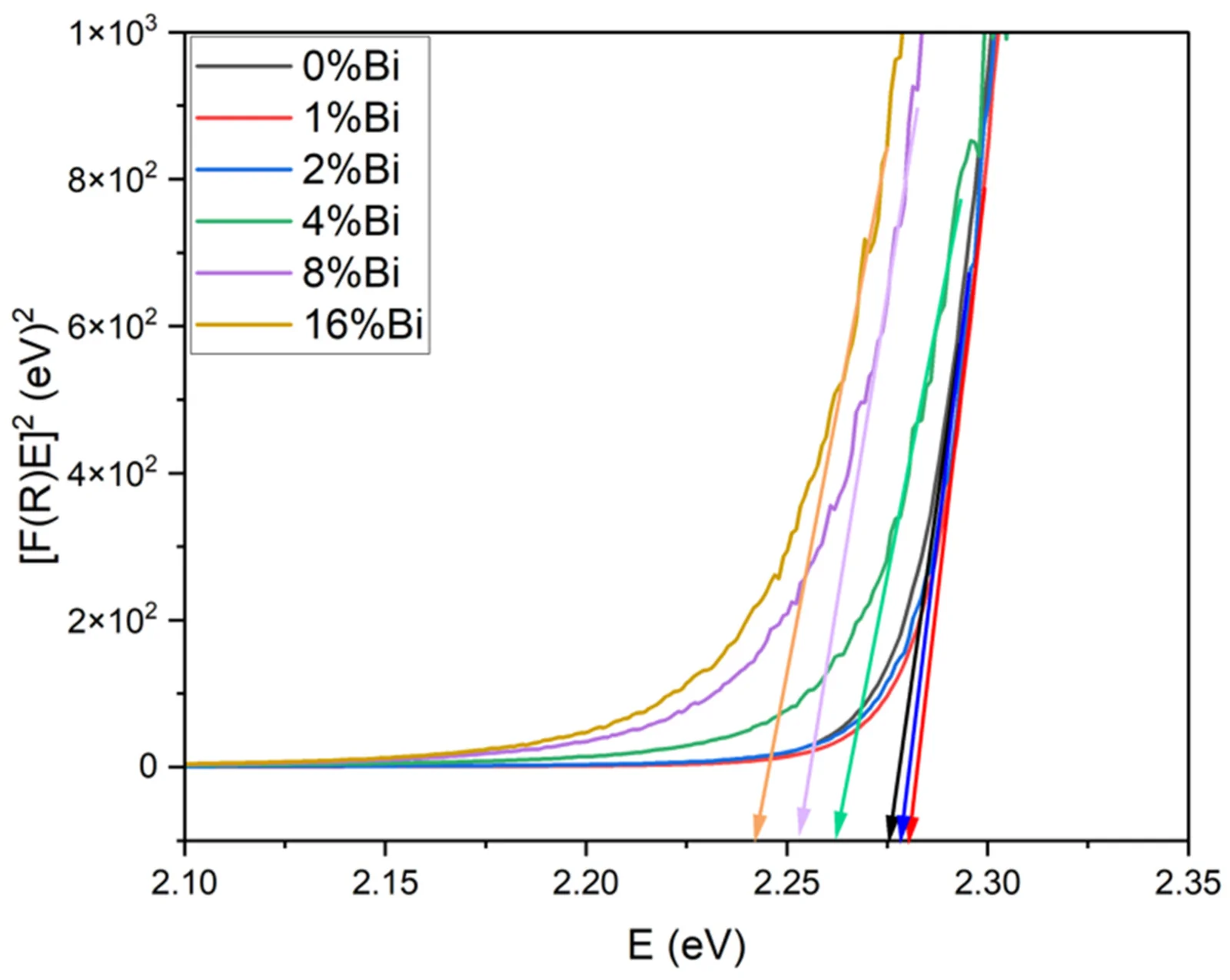
Associated Tauc plots (F(R)·E)^2^ as a function of photon energy for the MAPb_1−x_Bi_x_Br_3_ powder series, illustrating the linear extrapolation used to extract the values of the effective direct optical gaps.

**Figure 7:**
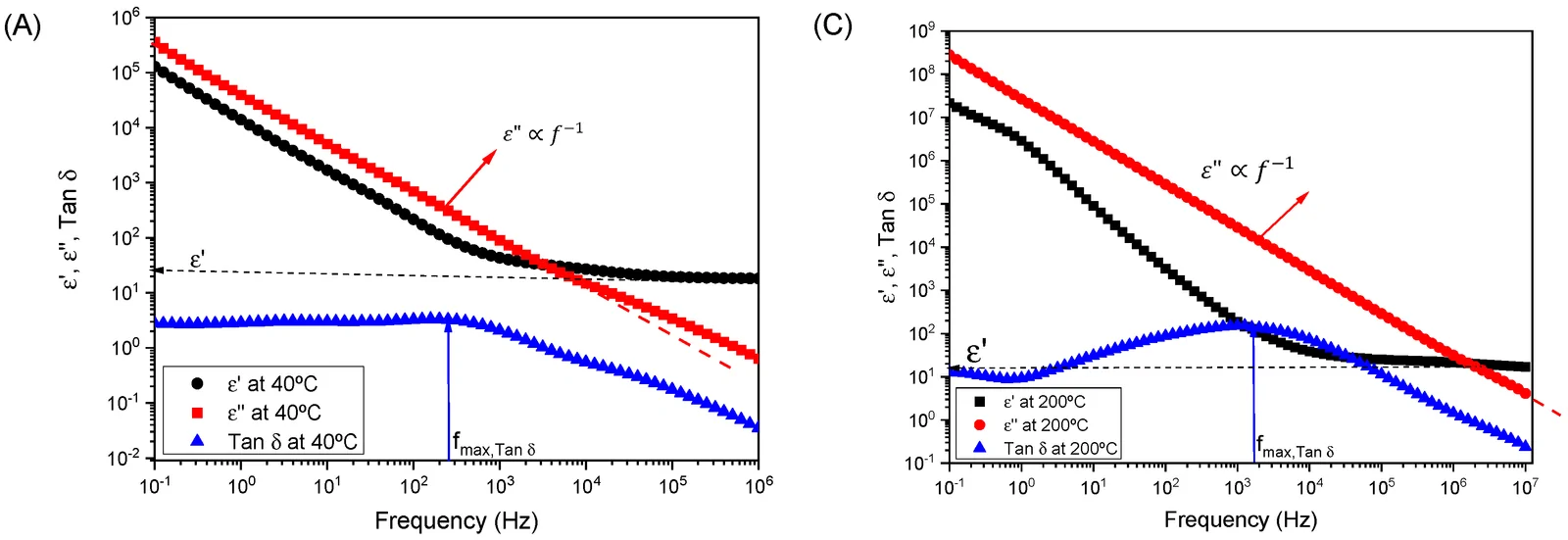

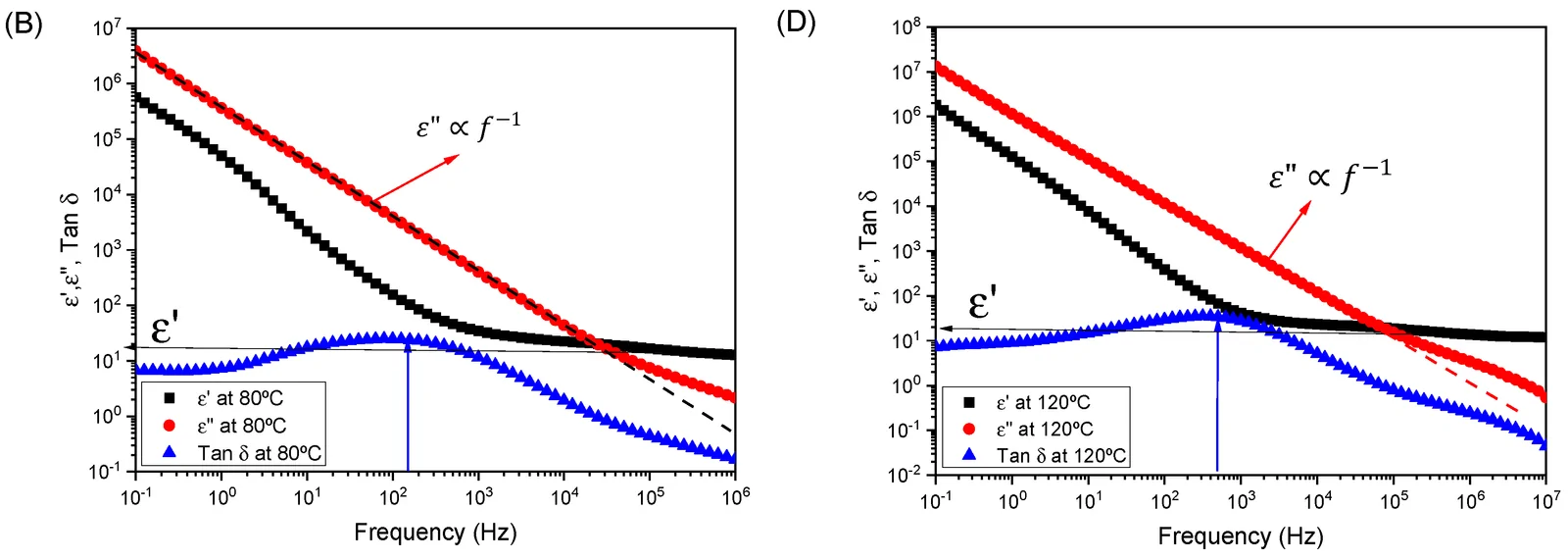
Dielectric permittivity ε′ (black), loss permittivity ε″ (red) and loss tangent Tan δ (blue) as a function of frequency for the sample MAPb_1−x_Bi_x_Br_3_ (x = 0.04) at (**A**) 40 °C, (**B**) 80 °C, (**C**) 120 °C and (**D**) 200 °C.

**Figure 8:**
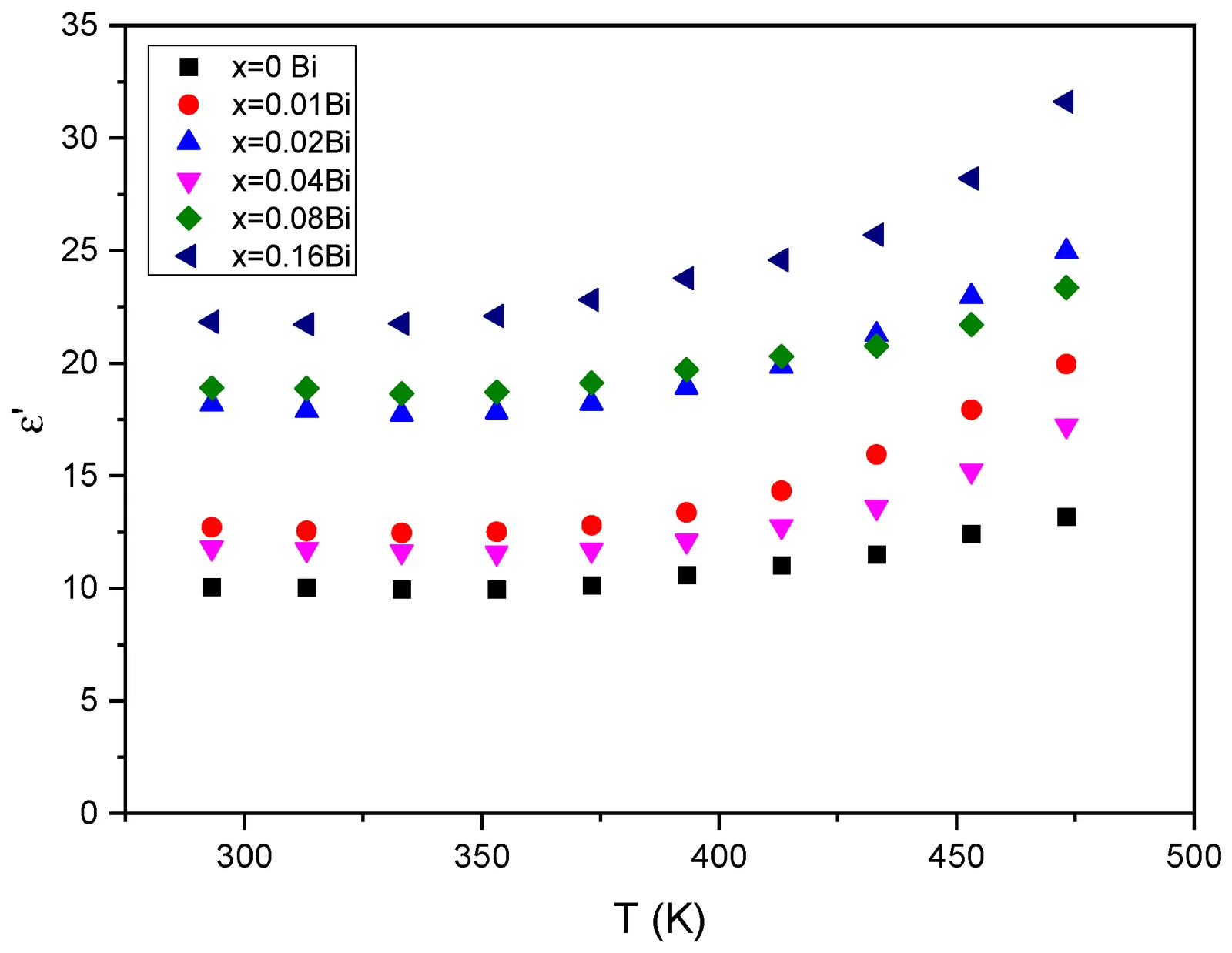
Temperature variation in the relative dielectric permittivity at f ⟶ ∞, for all the samples studied.

**Figure 9:**
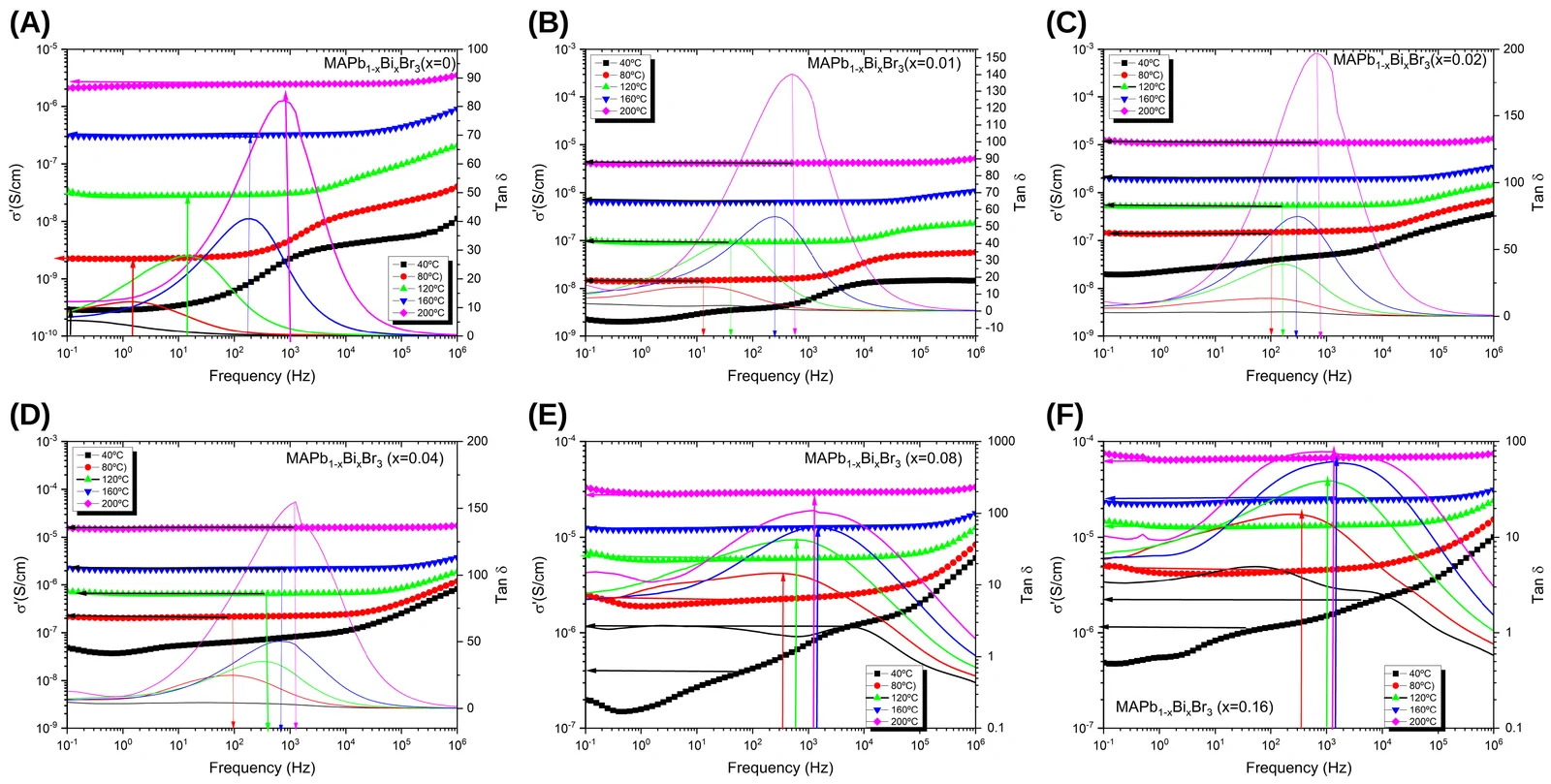
Real part of conductivity and loss tangent Tan δ at 40 °C, 80 °C, 120 °C, 160 °C and 200 °C, for the samples MAPb_1−x_Bi_x_Br_3_ (x = (**A**) 0, (**B**) 0.01, (**C**) 0.02, (**D**) 0.04, (**E**) 0.08 and (**F**) 0.16). The arrows allow us to observe the tan δ peak frequency and the corresponding dc-conductivity measured for each sample and temperature.

**Figure 10:**
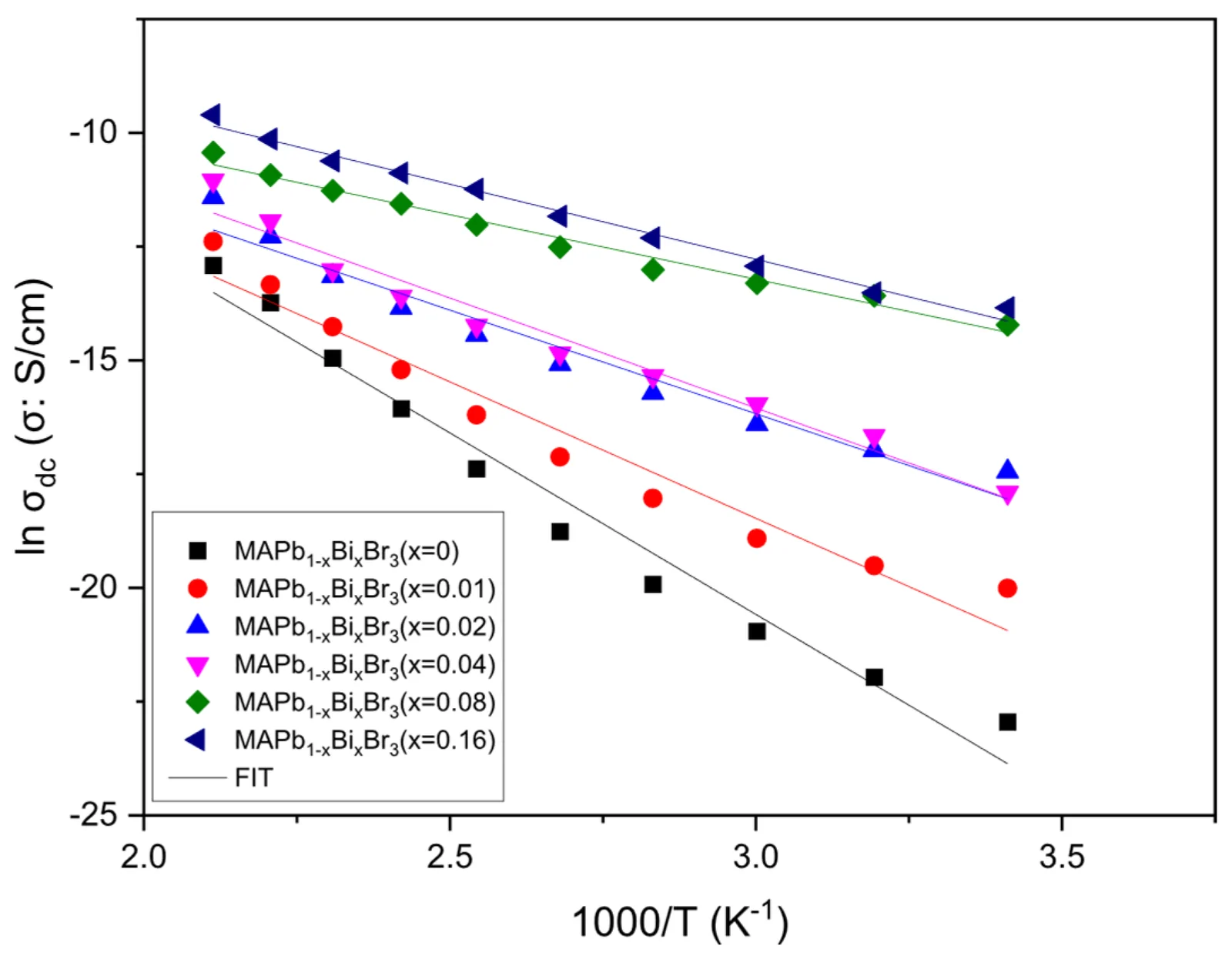
Arrhenius plot for the conductivity of the MAPb_1−x_Bi_x_Br_3_ powders with nominal bismuth (Bi) concentrations of x = 0, 0.01; 0.02; 0.04; 0.08 and 0.16, respectively. The lines represent the fits in all the range of temperatures.

**Figure 11:**
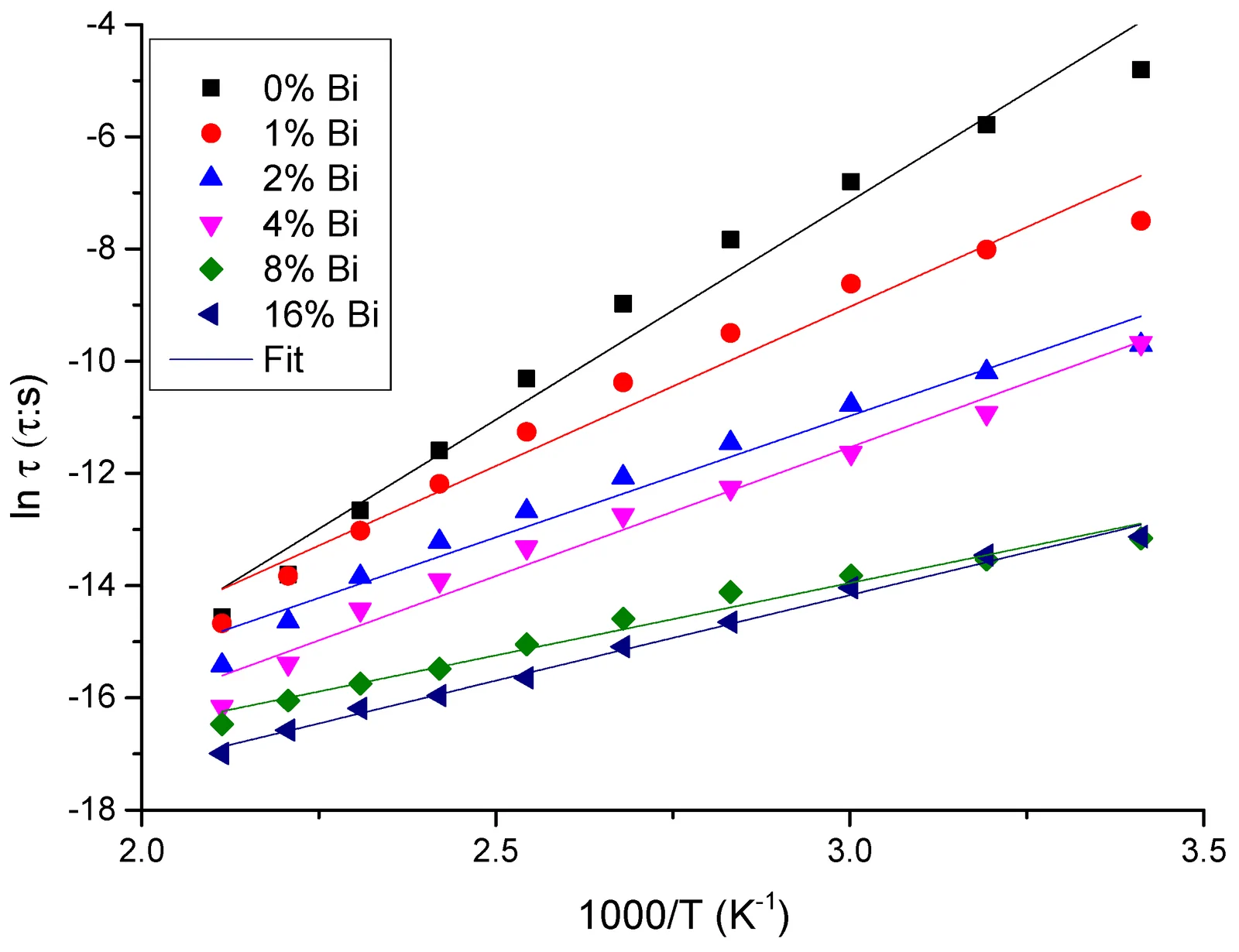
Arrhenius plot for the relaxation time associated with the conductivity for the powders MAPb_1−x_Bi_x_Br_3_ (x = 0, 0.01, 0.02, 0.04, 0.08, and 0.16 of Bi).

**Figure 12:**
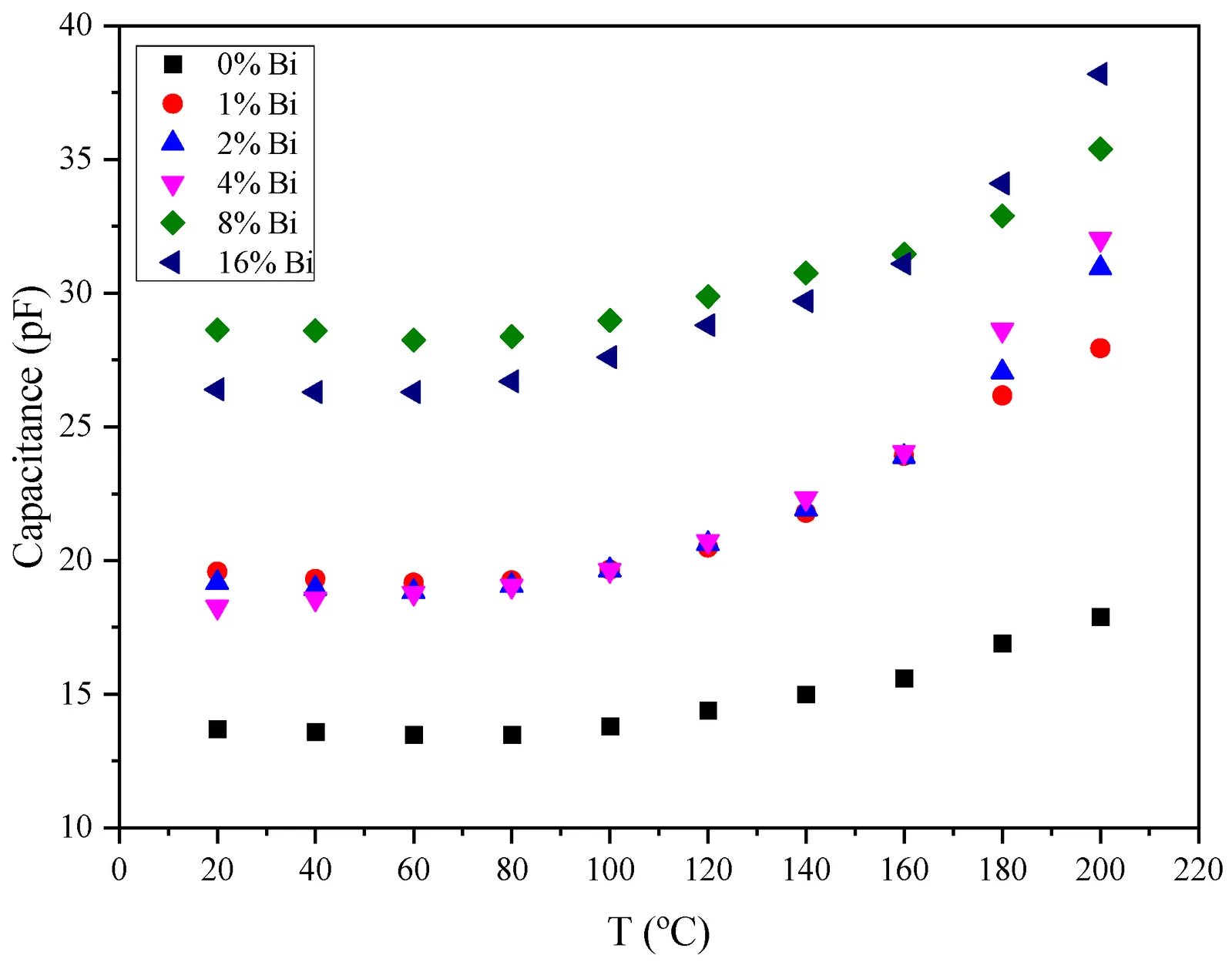
Apparent geometrical capacitance as a function of the temperature for the powders MAPb_1−x_Bi_x_Br_3_ (x = 0, 0.01, 0.02, 0.04, 0.08 and 0.16 of Bi).

**Table 1:** Lattice parameters and unit-cell volumes obtained from Rietveld refinement, together with the FWHM and absolute maximum intensity of the (100) diffraction peak extracted from the experimental XRD patterns. The formal esd (GSAS-II) of all lattice parameters is ±0.00001 Å.

Bi Content (%)	Lattice Parameter *a* (Å)	Unit-Cell Volume *V* (Å^3^)	Peak FWHM (100) (°)	Peak Intensity (a.u.)
0	5.92173	207.66	0.0934	559,117
1	5.92664	208.17	0.0934	762,750
2	5.92574	208.07	0.1080	859,646
4	5.92459	207.96	0.1071	1,218,042
8	5.92633	208.14	0.0888	928,579
16	5.92576	208.08	0.1001	884,521

**Table 2:** Effective optical band gap energies (Eg) extracted from the Tauc (F(R)·E)^2^ plots of the MAPb_1−x_Bi_x_Br_3_ powder series as a function of the nominal bismuth doping rate.

Nominal Sample (x)	Effective Optical Gap Energy Eg (eV)
0%	2.278 ± 0.005
1%	2.282 ± 0.005
2%	2.280 ± 0.005
4%	2.264 ± 0.005
8%	2.253 ± 0.005
16%	2.243 ± 0.005

## Data Availability

The original contributions presented in this study are included in the article/Supplementary Materials. Further inquiries can be directed to the corresponding author.

## Supplementary Materials

The following supporting information can be downloaded at: https://www.mdpi.com/article/10.3390/nano16201319/s1, Figure S1: Arrhenius plot for the conductivity of the MAPb_1−x_Bi_x_Br_3_ powders with nominal bismuth (Bi) concentrations of x = 0, 0.01, 0.02, 0.04, 0.08 and 0.16, respectively. The lines represent the fit to experimental data following two different behaviors; Figure S2: Arrhenius plot of apparent geometrical capacitance of MAPb_1−x_Bi_x_Br_3_ powders with nominal bismuth (Bi) concentrations of x = 0, 0.01, 0.02, 0.04, 0.08 and 0.16. respectively. The lines represent the fit to experimental data following two different behaviors. one at low temperatures and other for high temperatures; Table S1: Values of the activation energy obtained for the conductivity considering two different Arrhenius behavior in the range temperatures studied; Table S2: Values of the relaxation time τ_EP_ and τ of obtained from Equations (10) and (11) for all the perovskites powders. τ_m_ is the time obtained at the corresponding peak of the loss tangent for each temperature.
