# Supplementary material for: Solubility Limit and Optoelectronic Evolution of Heterovalent Bi3+ Doping in MAPbBr3 Perovskite Powders: A Study from Electrochemical Impedance Spectroscopy (EIS)
Source: Nanomaterials (Basel). 2026 Oct 19;16(20):1319. doi: 10.3390/nano16201319 (PMC13707914; doi:10.3390/nano16201319)
Supplement: Supplementary file 1 [file nanomaterials-16-01319-s001.zip › nanomaterials-4573196-supplementary.pdf]

# Solubility limit and optoelectronic evolution of heterovalent $Bi^{3+}$ doping in $MAPbBr_3$ perovskite powders: A study from Electrochemical Impedance Spectroscopy (EIS)

Péyèna Yacouba Dao[1], Andreu Andrio[2], Julia Marí-Guaita[1,*], Giulia Longo[1], Shafi Ullah[1], Donafologo Soro[3], Álvaro Montero Reguera[4], Bernabé Marí Soucase[1], Vicente Compañ[4]

[1]Universitat Politècnica de Valencia, Institut de Fabricació i Producció Automatitzada, Camí de Vera s/n, València, 46022, Spain

[2]Universitat Jaume I. Avinguda Sos Baynat s/n. Castellón 12071, Spain.

[3]Departement des Sciences et Technologie, Ecole Normale Supérieure (ENS) d'Abidjan, BP 10, Abidjan 08, Ivory Coast

[4]Departamento de Termodinámica Aplicada (ETSII), Universitat Politècnica de València, Camí de Vera s/n, 46022, València, Spain

Corresponding author: jumagu@upv.es

## Supplementary information

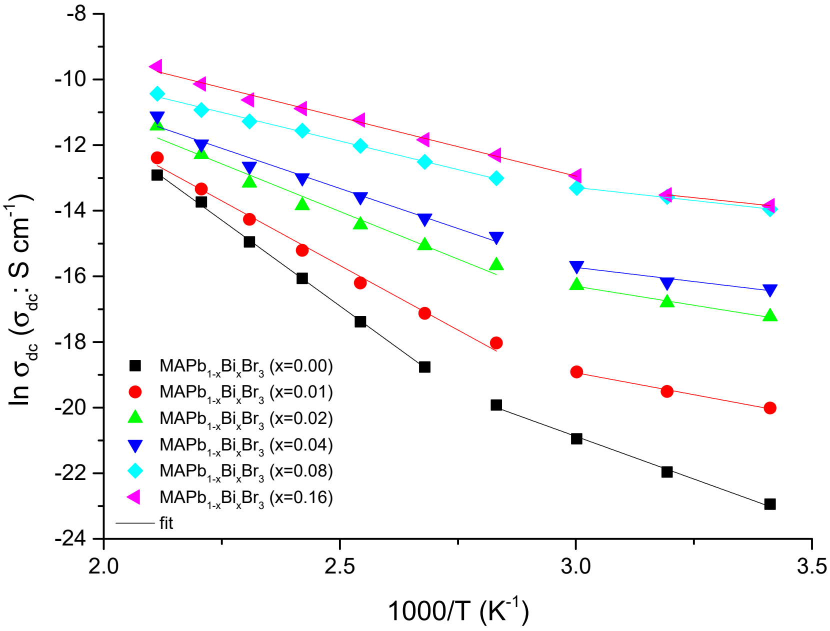


**Figure S1.** Arrhenius plot for the conductivity of the $MAPb_{1-x}Bi_xBr_3$ powders with nominal bismuth (Bi) concentrations of x = 0, 0.01; 0.02; 0.04; 0.08 and 0.16, respectively. The lines represent the fit to experimental data following two different behaviors.

In table S1 are tabulated the activation energies calculated in the two range of temperatures observed in the complete range studied.

**Table S1.** Values of the activation energy obtained for the conductivity considering two different Arrhenius behavior in the range temperatures studied.

| **Perovskite powders** | **x** | **$E_{act}$ (kJ/mol) [20ºC-80ºC]** | **$E_{act}$ (kJ/mol) [100ºC-200ºC]** |
|---|---|---|---|
| $MAPb_{1-x}Bi_xBr_3$ | **0.00** | **43.2 (0.448 eV)** | **86.8 (0.900 eV)** |
| $MAPb_{1-x}Bi_xBr_3$ | **0.01** | **22.3 (0.231 eV)** | **65.4 (0.678 eV)** |
| $MAPb_{1-x}Bi_xBr_3$ | **0.02** | **19.2 (0.199 eV)** | **48.2 (0.500 eV)** |
| $MAPb_{1-x}Bi_xBr_3$ | **0.04** | **14.3 (0.148 eV)** | **40.6 (0.420 eV)** |
| $MAPb_{1-x}Bi_xBr_3$ | **0.08** | **13.4 (0.139 eV)** | **28.9 (0.300 eV)** |
| $MAPb_{1-x}Bi_xBr_3$ | **0.16** | **12.5 (0.129 eV)** | **27.4 (0.284 eV)** |

1kJ/mol = 0.010364eV.

**Table S2.** Values of the relaxation time $\tau_{EP}$ and $\tau$ of obtained from eq.(10) and (11) for all the perovskites powders. $\tau_m$ is the time obtained at the corresponding peak of the loss tangent for each temperature.

| **Perovskite Powder and Thickness** | **T (ºC)** | **$\tau_m$ (s)** | **$\tau_{EP}$ (s)** | **$\tau$ (s)** |
|---|---|---|---|---|
| **$MAPb_{1-x}Bi_xBr_3$ (x=0)** | 20 | | | 8.21E-03 |
| | 40 | | | 3.07E-03 |
| | 60 | 0.3180 | 90.842 | 1.11E-03 |
| **L** = 512 μm | 80 | 0.1000 | 25.500 | 3.95E-04 |
| | 100 | 0.0320 | 7.975 | 1.26E-04 |
| | 120 | 0.0100 | 3.035 | 3.32E-05 |
| | 140 | 0.0030 | 0.689 | 9.24E-06 |
| | 160 | 0.0010 | 0.200 | 3.17E-06 |
| | 180 | 0.0003 | 0.100 | 1.01E-06 |
| | 200 | 0.0002 | 0.085 | 4.75E-07 |

| **Perovskite Powder** | **T (ºC)** | **$\tau_m$ (s)** | **$\tau_{EP}$ (s)** | **$\tau$ (s)** |
|---|---|---|---|---|
| **$MAPb_{1-x}Bi_xBr_3$ (x=0.01)** | 20 | | | 0.00055296 |
| | 40 | | | 0.00033110 |
| | 60 | 0.0500 | 1.42 | 0.00026824 |
| **L** = 446 μm | 80 | 0.0100 | 1.34 | 0.00007514 |
| | 100 | 0.0100 | 0.82 | 0.00003106 |
| | 120 | 0.0040 | 0.79 | 0.00001286 |
| | 140 | 0.0020 | 0.50 | 0.00000510 |
| | 160 | 0.0010 | 0.18 | 0.00000221 |
| | 180 | 0.0004 | 0.16 | 0.00000099 |
| | 200 | 0.0003 | 0.24 | 0.00000042 |

| **Perovskite Powder** | **T (ºC)** | **$\tau_m$ (s)** | **$\tau_{EP}$ (s)** | **$\tau$ (s)** |
|---|---|---|---|---|

| | | | | |
|---|---|---|---|---|
| **$MAPb_{1-x}Bi_xBr_3$ (x=0.02)** | 20 | 0.00100 | 0.02 | 6.06E-05 |
| | 40 | 0.00115 | 0.04 | 3.74E-05 |
| **L** = 669 μm | 60 | 0.00126 | 0.08 | 2.09E-05 |
| | 80 | 0.00252 | 0.60 | 1.06E-05 |
| | 100 | 0.00200 | 0.70 | 5.74E-06 |
| | 120 | 0.00100 | 0.32 | 3.13E-06 |
| | 140 | 0.00080 | 0.35 | 1.82E-06 |
| | 160 | 0.00050 | 0.26 | 9.73E-07 |
| | 180 | 0.00032 | 0.23 | 4.39E-07 |
| | 200 | 0.00025 | 0.32 | 2.01E-07 |

| **Perovskite Powder** | **T (°C)** | **$\tau_m$ (s)** | **$\tau_{EP}$ (s)** | **τ (s)** |
|---|---|---|---|---|
| **$MAPb_{1-x}Bi_xBr_3$ (x=0.04)** | 20 | | | 6.25E-05 |
| | 40 | 0.00634 | 2.24 | 1.79E-05 |
| **L** = 471 μm | 60 | 0.00318 | 1.14 | 8.85E-06 |
| | 80 | 0.00159 | 0.54 | 4.73E-06 |
| | 100 | 0.00080 | 0.22 | 2.91E-06 |
| | 120 | 0.00050 | 0.15 | 1.64E-06 |
| | 140 | 0.00032 | 0.11 | 9.10E-07 |
| | 160 | 0.00025 | 0.12 | 5.43E-07 |
| | 180 | 0.00020 | 0.19 | 2.07E-07 |
| | 200 | 0.00013 | 0.17 | 9.61E-08 |

| **Perovskite Powder** | **T (°C)** | **$\tau_m$ (s)** | **$\tau_{EP}$ (s)** | **τ (s)** |
|---|---|---|---|---|
| **$MAPb_{1-x}Bi_xBr_3$ (x=0.08)** | 20 | | | 1.93E-06 |
| | 40 | 0.06336 | 3041.44 | 1.32E-06 |
| **L** = 459 μm | 60 | 0.00634 | 40.49 | 9.92E-07 |
| | 80 | 0.00063 | 0.54 | 7.43E-07 |
| | 100 | 0.00050 | 0.55 | 4.60E-07 |
| | 120 | 0.00032 | 0.35 | 2.92E-07 |
| | 140 | 0.00013 | 0.08 | 1.89E-07 |
| | 160 | 0.00013 | 0.11 | 1.45E-07 |
| | 180 | 0.00013 | 0.15 | 1.07E-07 |
| | 200 | 0.00013 | 0.23 | 7.03E-08 |

| **Perovskite Powder** | **T (°C)** | **$\tau_m$ (s)** | **$\tau_{EP}$ (s)** | **τ (s)** |
|---|---|---|---|---|
| **$MAPb_{1-x}Bi_xBr_3$ (x=0.16)** | 20 | | | 1.99E-06 |
| | 40 | 0.00318 | 7.06 | 1.43E-06 |
| **L** = 575 μm | 60 | 0.00252 | 7.97 | 7.98E-07 |
| | 80 | 0.00063 | 0.92 | 4.34E-07 |
| | 100 | 0.00050 | 0.91 | 2.79E-07 |
| | 120 | 0.00016 | 0.16 | 1.61E-07 |
| | 140 | 0.00016 | 0.22 | 1.17E-07 |

| | | | | |
|---|---|---|---|---|
| | 160 | 0.00016 | 0.27 | 9.35E-08 |
| | 180 | 0.00016 | 0.40 | 6.33E-08 |
| | 200 | 0.00016 | 0.61 | 4.17E-08 |

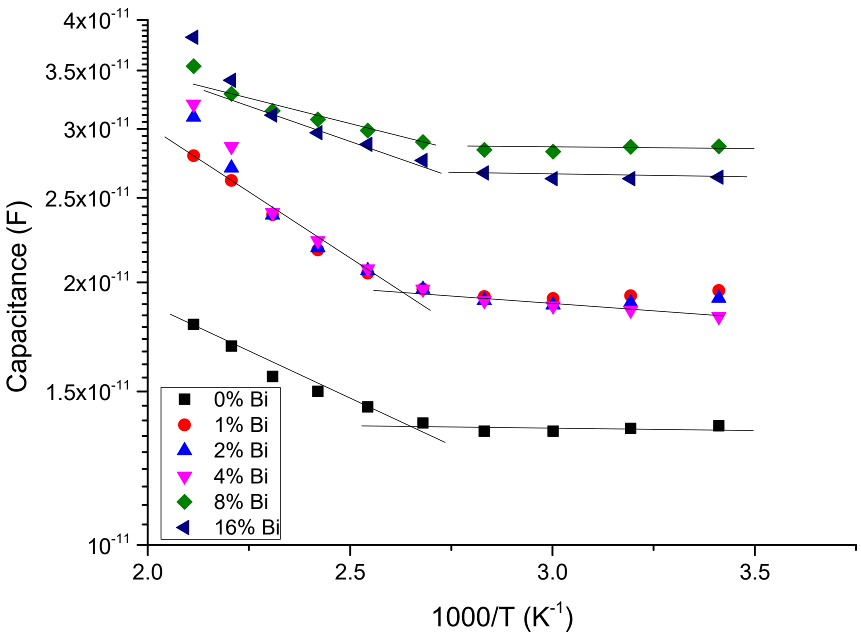


**Figure S2.** Arrhenius plot of apparent geometrical capacitance of $MAPb_{1-x}Bi_xBr_3$ powders with nominal bismuth (Bi) concentrations of x = 0; 0.01; 0.02; 0.04; 0.08 and 0.16. respectively. The lines represent the fit to experimental data following two different behaviors. one at low temperatures and other for high temperatures.
